# Supplementary material for: Spatial genomic heterogeneity in multiple myeloma revealed by multi-region sequencing
Source: Nat Commun. 2017 Aug 16;8:268. doi: 10.1038/s41467-017-00296-y (PMC5559527; doi:10.1038/s41467-017-00296-y)
Supplement: Supplementary file 1 — Supplementary Information [file 41467_2017_296_MOESM1_ESM.pdf]

File name: Supplementary Information
Description: Supplementary Figures and Supplementary Table

File name: Supplementary Data 1
Description: Sample origin and purity

File name: Supplementary Data 2
Description: Shared and unshared copy number aberrations

File name: Supplementary Data 3
Description: Number of non-silent mutations

File name: Supplementary Data 4
Description: Previous therapies for “Treated patients”

File name: Supplementary Data 5
Description: Identification of evolutionary dynamics in multiple myeloma

File name: Supplementary Data 6
Description: Confirmation of unshared mutations by deep whole exome sequencing

File name: Peer Review File
Description:

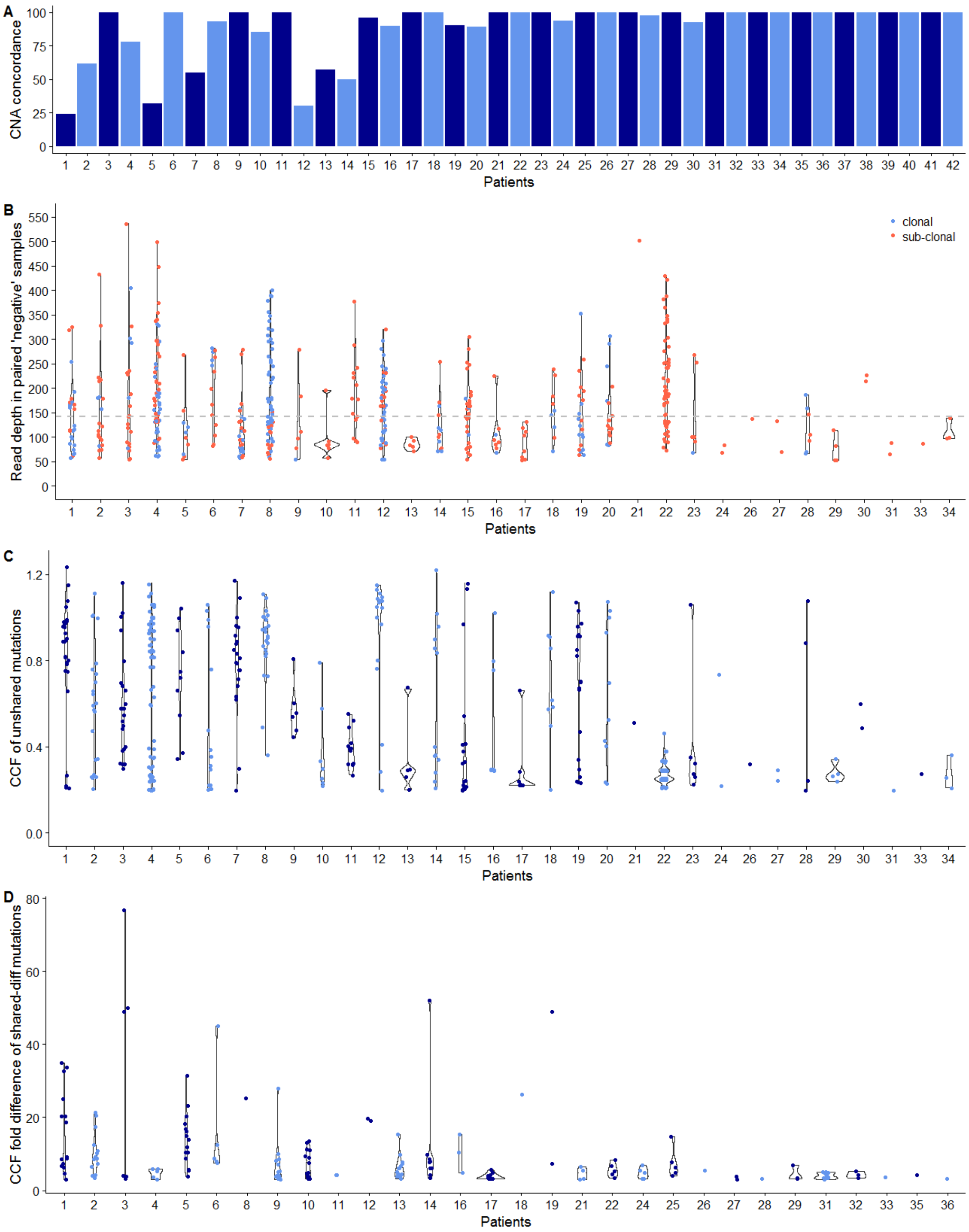

Supplementary Figure 1. The figure presents the concordance for copy number aberrations between paired samples **(A)**, the read depth at the sites of unshared mutations in the paired negative sample **(B)**, the cancer clonal fraction (CCF) of unshared mutations **(C)**, and the CCF difference between paired samples for shared-diff mutations **(D)**.

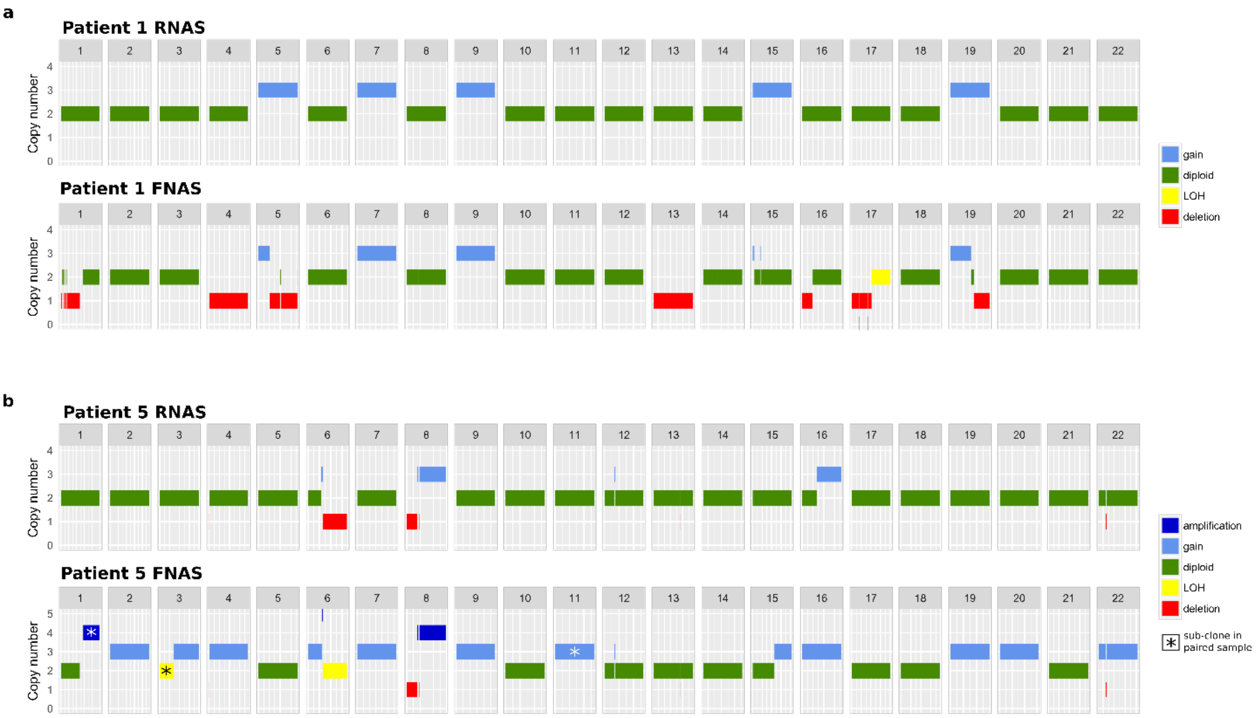

Supplementary Figure 2: Chromosomal profiles. The plot illustrates the chromosomal profiles for paired samples of patient #1 and #5 who presented with discordance regarding the ploidy status.

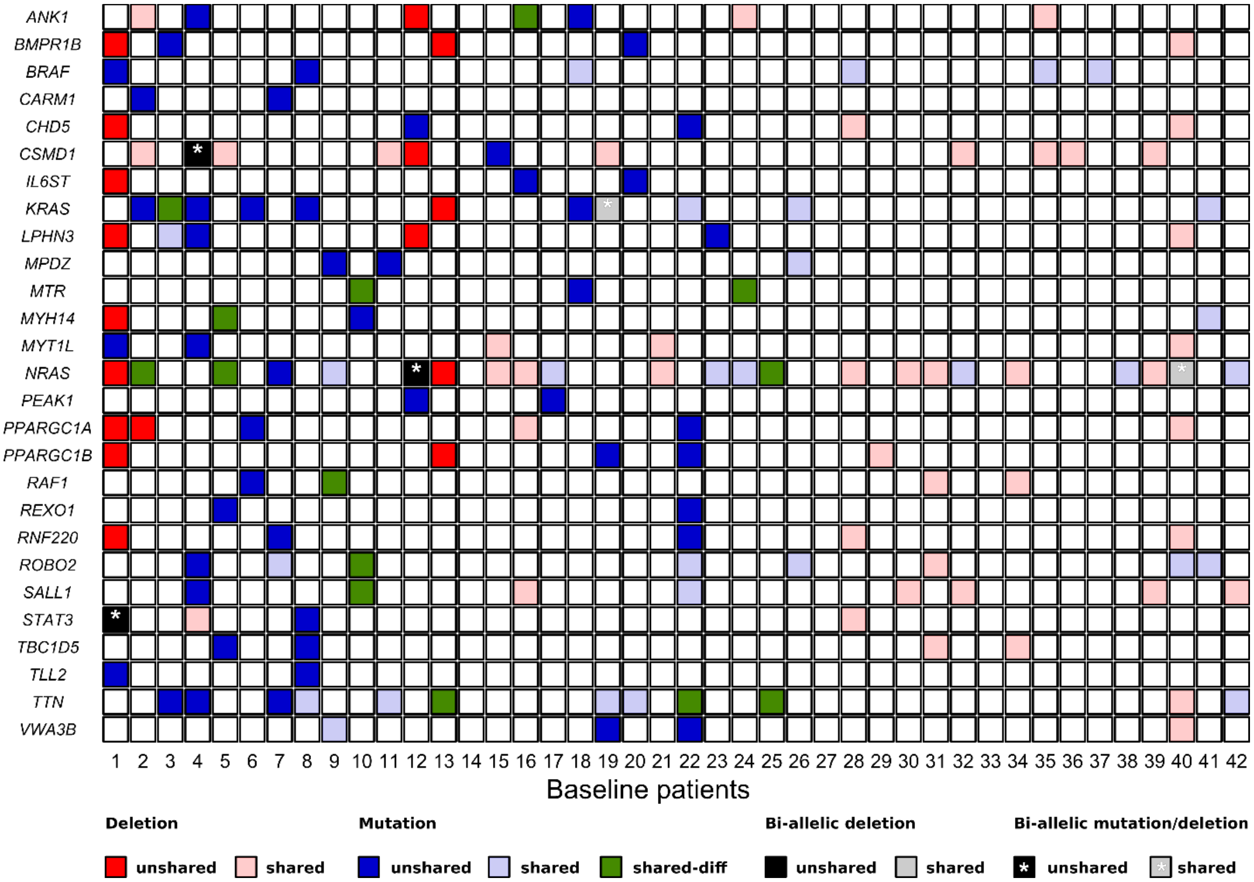

Supplementary Figure 3: Genes most frequently showing heterogeneity in baseline patients. The plot shows non-silent mutations and deletions for genes that most often presented with spatial heterogeneity in 42 newly diagnosed patients. Blue denotes mutations, red deletions and black bi-allelic events. Light colors indicate shared events. * Mutation & deletion or mutation and loss of heterozygosity

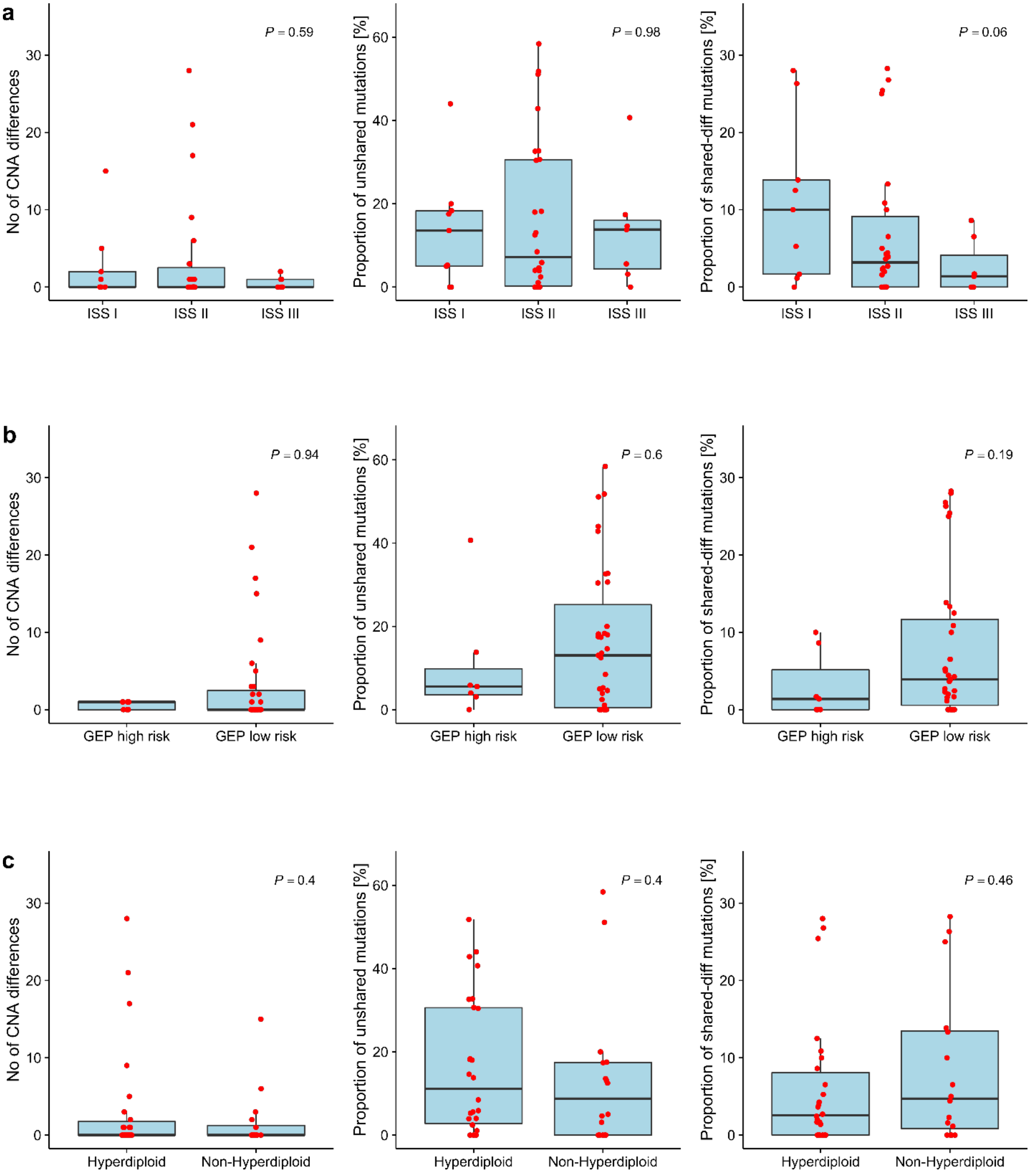

Supplementary Figure 4: Spatial heterogeneity in baseline patients stratified by standard clinical and molecular variables. Copy number aberration differences (CNA) and the proportion of *unshared* and *shared-diff* non-silent mutations for 42 newly diagnosed patients stratified by **(a)** the ISS stage, **(b)** the GEP70 risk score, and **(c)** the ploidy status. P-values correspond to univariate statistics for linear regression (ISS) or Wilcoxon tests not corrected for multiple testing.

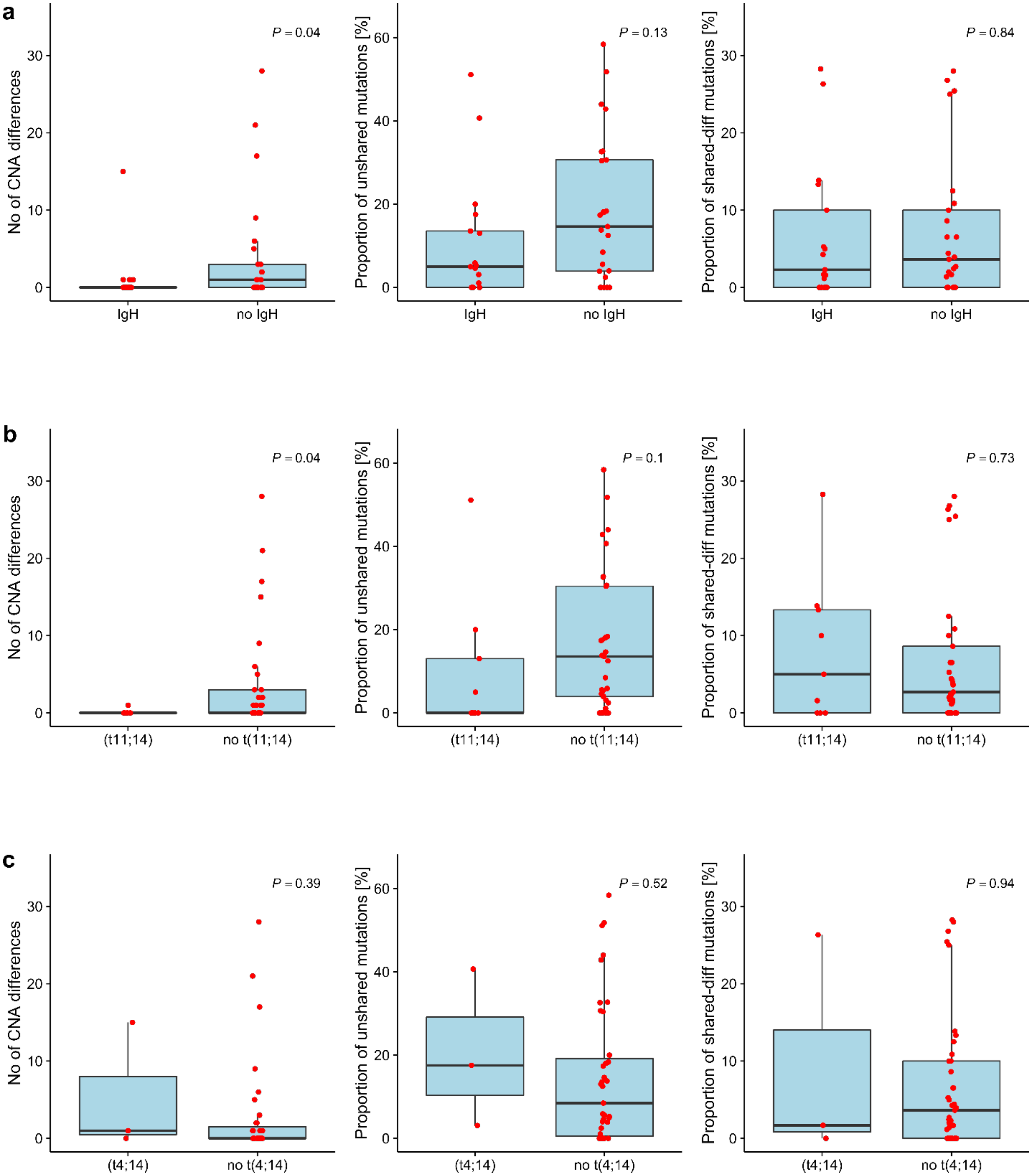

Supplementary Figure 5: Spatial heterogeneity in baseline patients stratified by standard clinical and molecular variables. Spatial heterogeneity in 42 newly diagnosed patients stratified by **(a)** the presence of primary recurrent IgH translocations, **(b)** the presence of a primary t(11;14) translocation, and **(c)** the presence of a primary t(4;14) translocation. P-values correspond to univariate statistics for Wilcoxon tests not corrected for multiple testing.

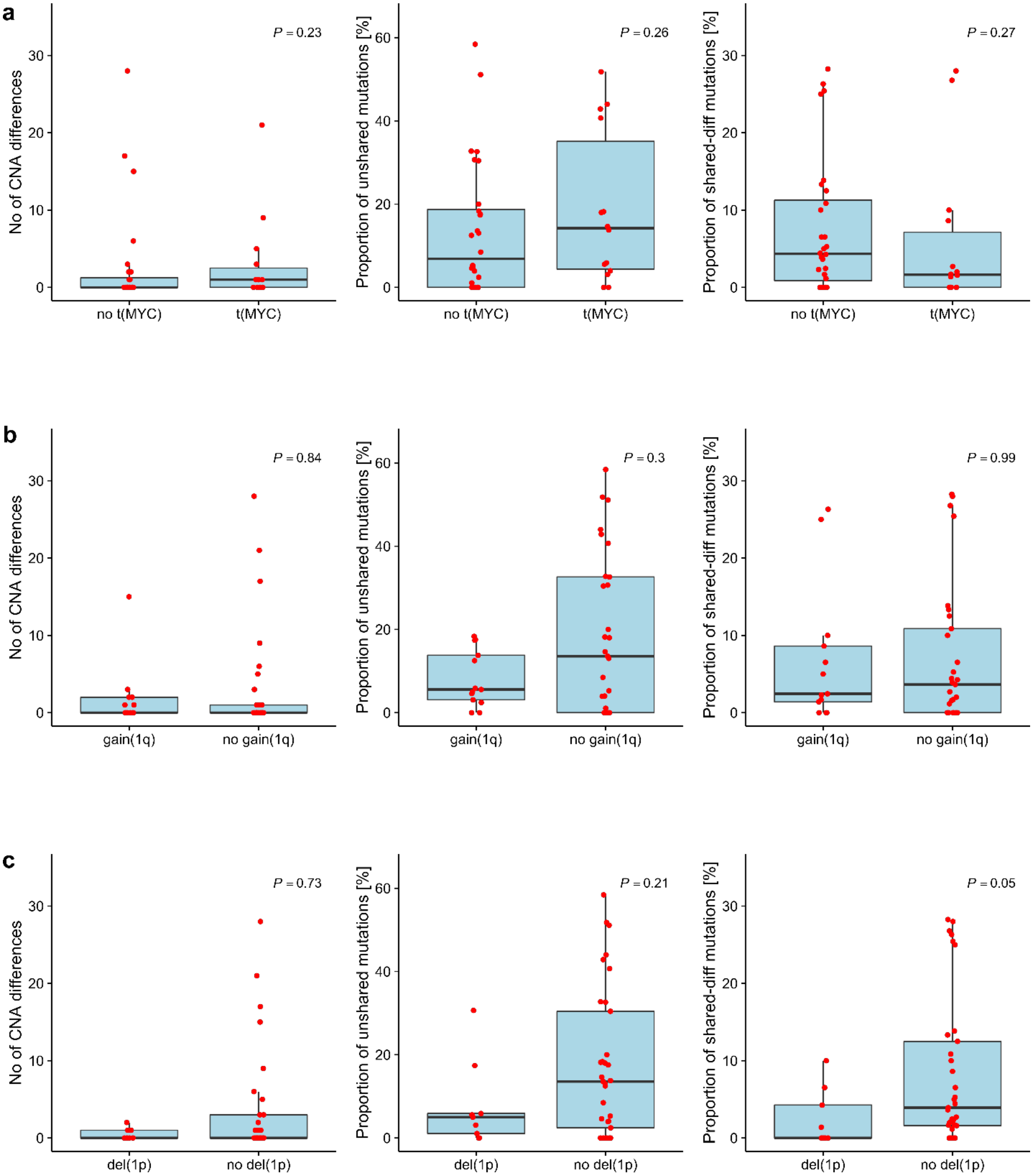

Supplementary Figure 6: Spatial heterogeneity in baseline patients stratified by standard clinical and molecular variables. Spatial heterogeneity in 42 newly diagnosed patients stratified by **(a)** the presence of translocations involving the MYC locus (t(MYC)), **(b)** the presence of gain1q, and **(c)** the presence of deletions involving 1p. P-values correspond to univariate statistics for Wilcoxon tests not corrected for multiple testing.

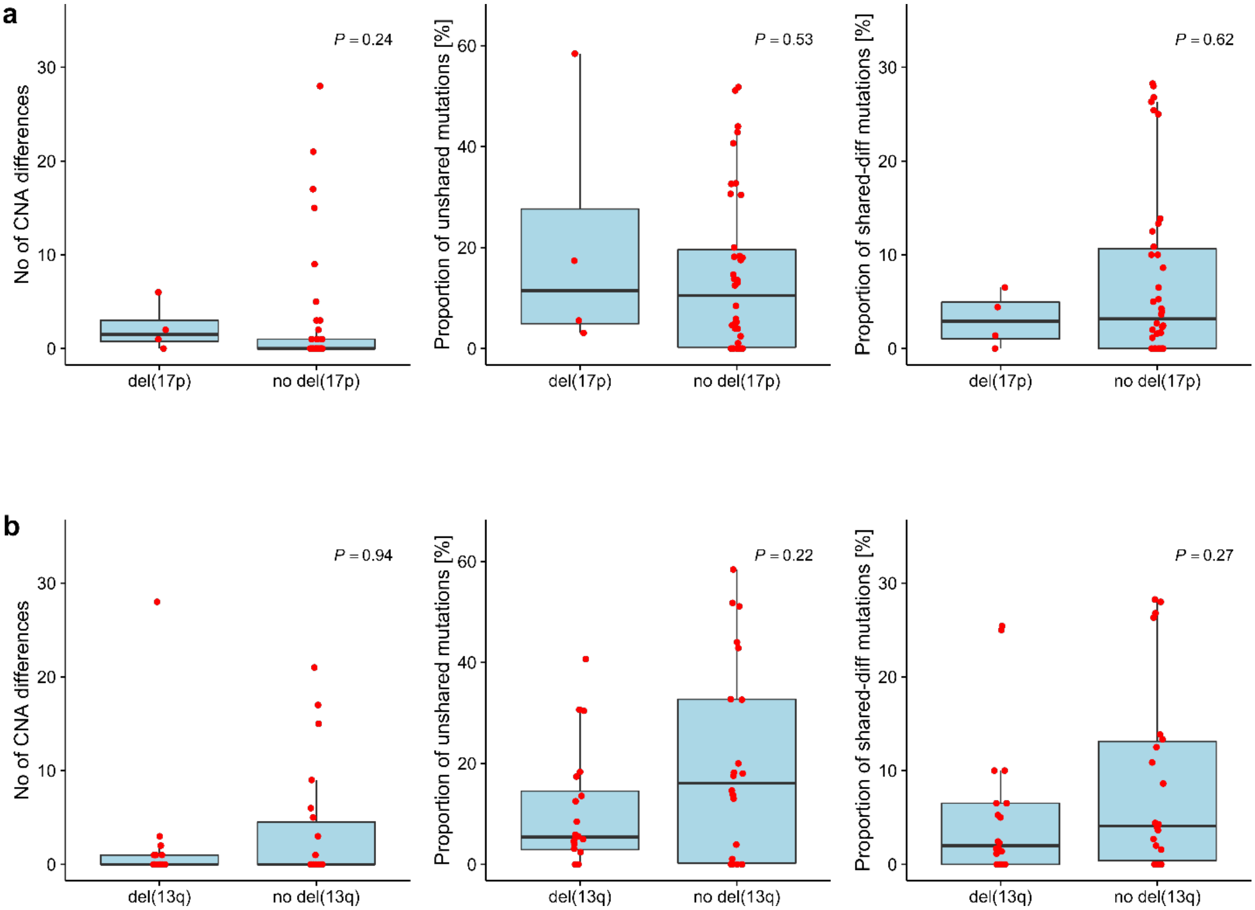

Supplementary Figure 7: Spatial heterogeneity in baseline patients stratified by standard clinical and molecular variables. Spatial heterogeneity in 42 newly diagnosed patients stratified by **(a)** the presence of deletions involving 17p12, and **(b)** the presence of deletions involving 13q14. P-values correspond to univariate statistics for Wilcoxon tests not corrected for multiple testing.

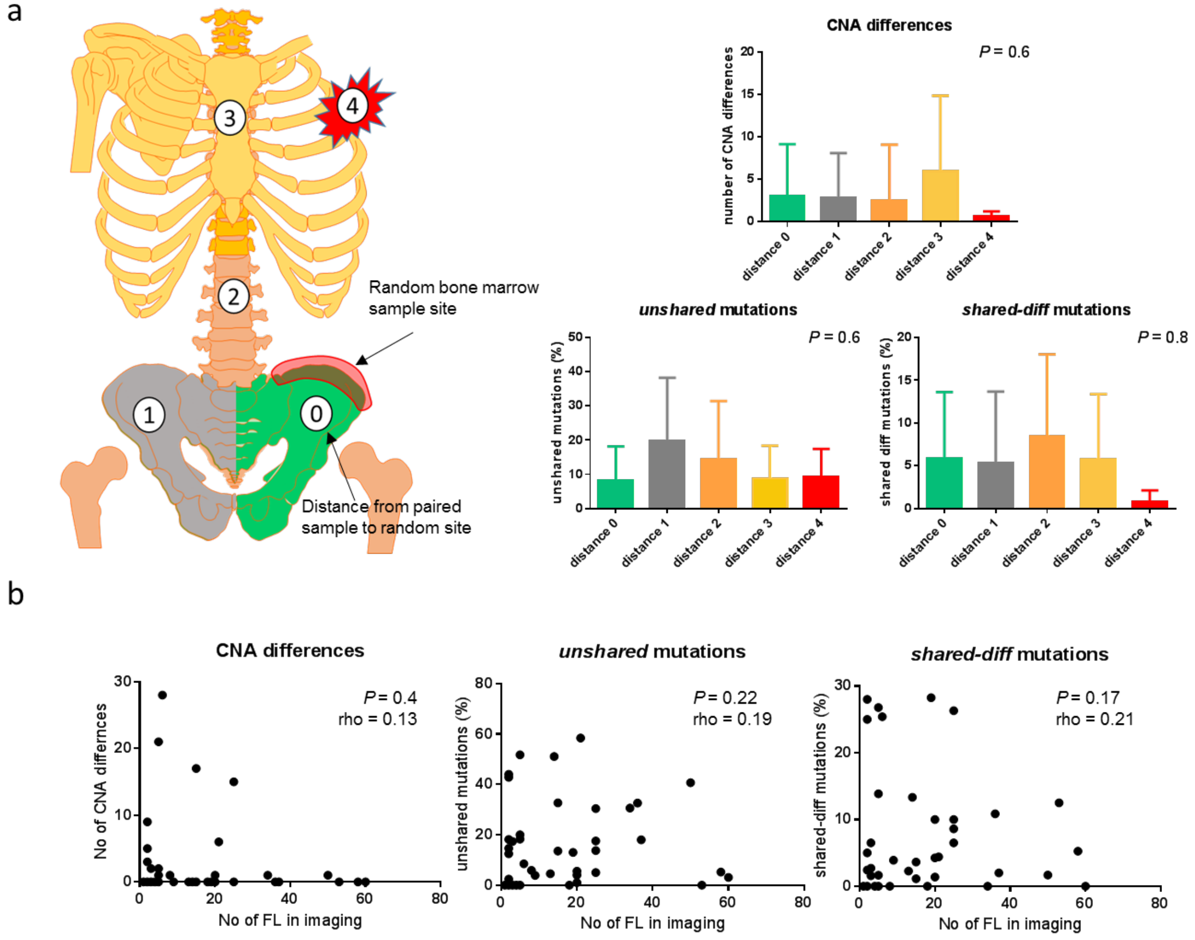

Supplementary Figure 8: Association between spatial heterogeneity in baseline patients and the anatomical distance between investigated sites or the total number of focal lesions. In **(a)** the anatomical distance between CT-guided sites and the random iliac crest was subdivided into five groups: Distance 0 comprised unilateral lesion from the pelvis where the random bone marrow aspiration had been taken from (n=12 patients). Sites at the contralateral pelvis were defined as distance 1 (n=21); distance 2 encompassed lesions from the lumbar spine (n=10), and all other skeletal lesions were assigned to distance 3 (n=11). Extra-medullary disease was classified as distance 4 (n=3). The plot shows the level of heterogeneity stratified by anatomical distance. The association between the level of spatial heterogeneity and distance was tested by linear regression. The plot shows mean + standard deviation. In **(b)** correlation plots for spatial heterogeneity vs. the total number of PET-CT detectable focal lesions per patient are presented. The correlation coefficient was calculated using Spearman's rank correlation.

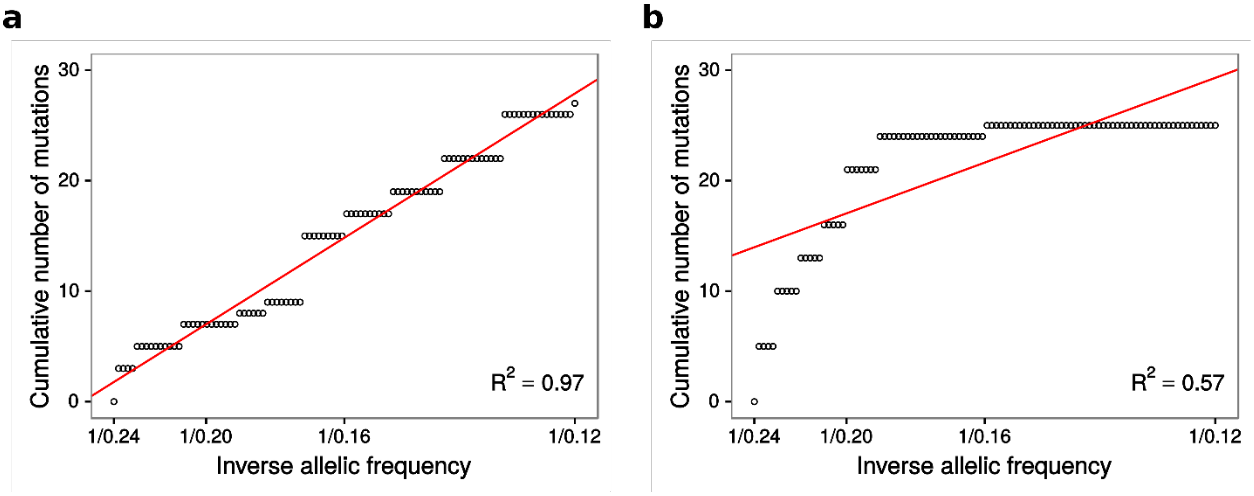

Supplementary Figure 9: Application of a *neutral* growth equation to multiple myeloma samples. None of the samples from newly diagnosed patients in our set presented with a variant allele frequency distribution characteristic for *neutral* growth ($R^2 \geq 0.98$). The plot shows the cumulative distribution of subclonal mutations vs the inverse of the corresponding variant allele frequency for (**a**) one patient with a high goodness-of-fit measure R^2 that nearly fulfilled the criteria for *neutral* growth and (**b**) for a sample with an enrichment of mutations at higher frequencies.

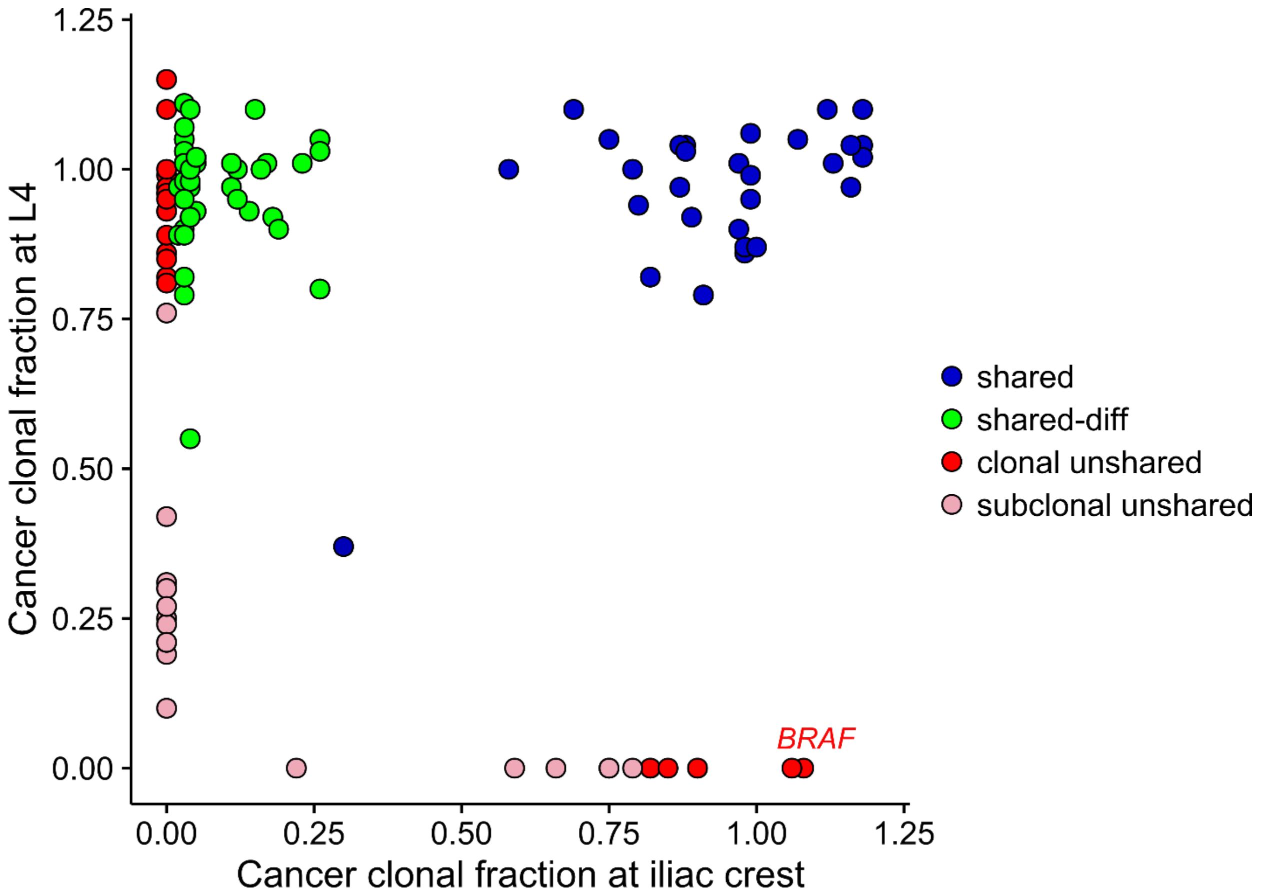

Supplementary Figure 10: Spatial clonal substructure. The cancer clonal fraction (CCF) of mutations detected in patient #1 at the iliac crest and a focal lesion at L4 is presented. Blue denotes shared mutations, *shared-diff* mutations are depicted in green. *Unshared* mutations are shown in red (CCF≥0.8) or light pink (CCF<0.8) to discriminate between clonal and sub-clonal mutations. The *unshared* clonal *BRAF* mutation found at the iliac crest is annotated.

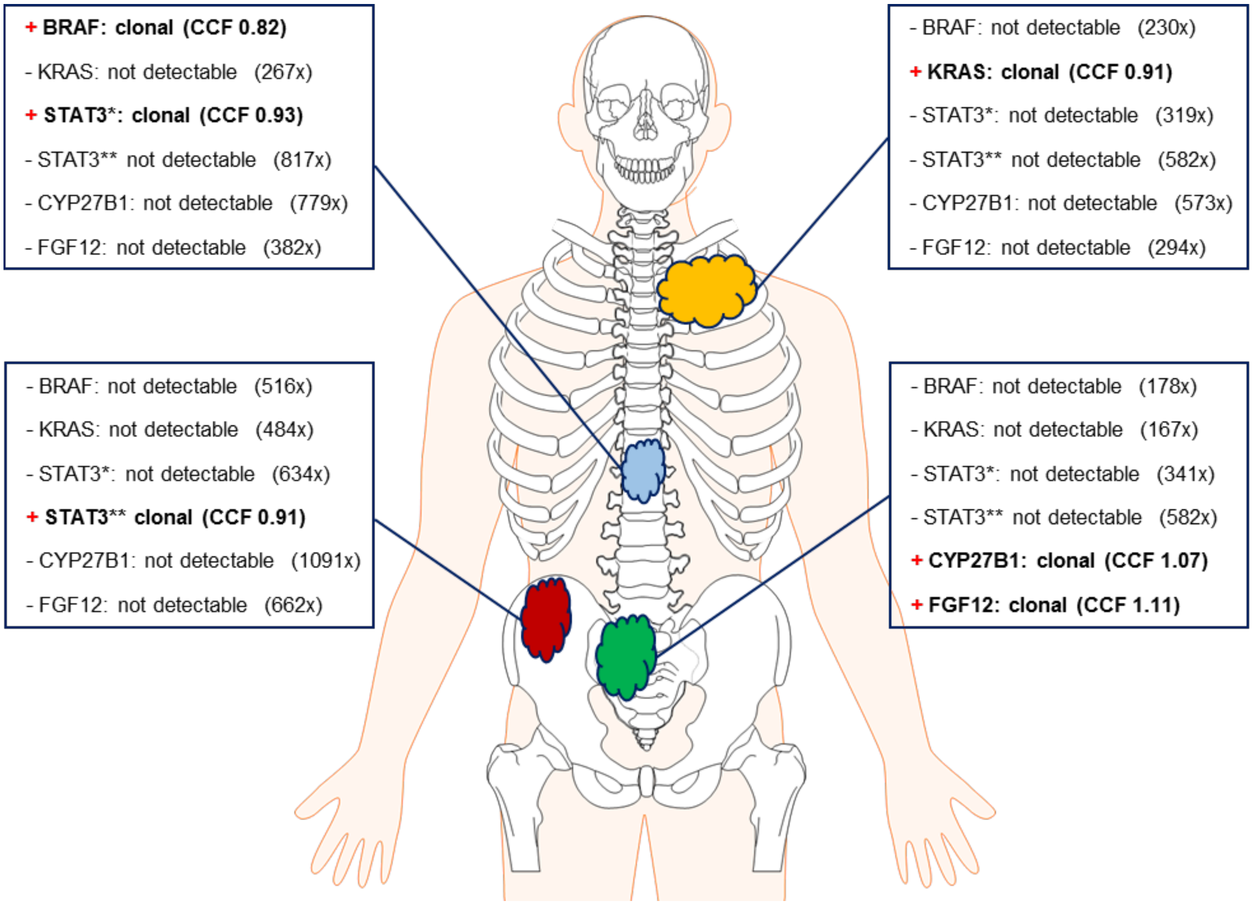

Supplementary Figure 11: Confirmation of unshared mutations at higher depth. The figure shows the results of deep WES sequencing for patient #8. The unshared non-silent mutations in the driver genes *BRAF*, *KRAS* and *STAT3* (*Asn553Lys, **Asp661Tyr) and the other genes *CYP27B1* and *FGF12* were selected as representative examples. The cancer clonal fraction (CCF) at the positive sites and the sequencing depth at the negative sites are shown in brackets. Please also see Supplementary Data 6 for a complete overview of deep WES confirmation of non-silent unshared mutations in patient #3, #7, #8, and #19.

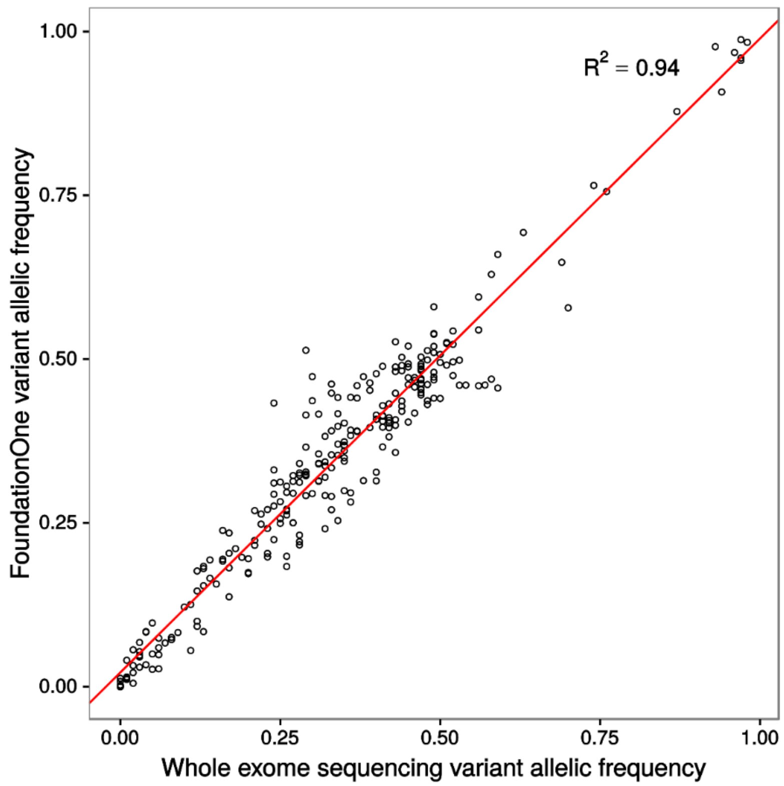

Supplementary Figure 12: Comparison of whole exome and targeted sequencing data. 53 samples of our whole exome sequencing study (31 patients) were also processed using the targeted sequencing F1H Panel (Foundation Medicine, MA) which covers 405 genes. The plot shows the F1H and whole exome sequencing variant allele frequency for mutations that were called by whole exome sequencing and were covered by the F1H Panel.

Supplementary Table 1: Patient characteristics.

		WES newly diagnosed (n=42)	WES treated (n=11)	Survival paired GEP70 (n=263)
Sex	Female	17 (40%)	4 (36%)	102 (39%)
Age	median (range)	63 (46-80)	67 (46-75)	61 (34-75)
ISS	I	9 (21%)	-	67 (25%)
	II	26 (62%)	-	119 (45%)
	III	7 (17%)	-	71 (27%)
	not available	0	-	5 (2%)
Ig type	IgA	10 (24%)	5 (45%)	44 (17%)
	IgG	24 (57%)	4 (36%)	164 (62%)
	IgD	0	0	2 (1%)
	IgM	0	0	1 (0%)
	Light chain MM	5 (12%)	2 (18%)	50 (19%)
	Nonsecretory	3 (7%)	0	2 (1%)
GEP70 (iliac crest)	high risk	7 (17%)	7 (64%)	42 (16%)

Abbreviations: WES: Whole exome sequencing, ISS: International staging system, Ig: Immunoglobulin
