# Supplementary material for: Spatial genomic heterogeneity in multiple myeloma revealed by multi-region sequencing
Source: Nat Commun. 2017 Aug 16;8:268. doi: 10.1038/s41467-017-00296-y (PMC5559527; doi:10.1038/s41467-017-00296-y)
Supplement: Supplementary file 2 — Supplementary Data 1 [file 41467_2017_296_MOESM2_ESM.pdf]

Supplementary Data 1: Sample origin and purity.

PID	Time point	Posterior iliac crest	FNAS 1	FNAS 2	FNAS 3	FNAS 4
1	Baseline	left (0.73)*	L4 (0.97)			
2	Baseline	left (0.85)	Ilium right (0.99)			
3	Baseline	right (0.8)	Symphysis pubis (1)			
4	Baseline	right (0.51)	Ilium left (0.62)			
5	Baseline	right (0.76)	Ilium right (0.94)			
6	Baseline	left (0.91)	Sacrum right (0.87)			
7	Baseline	right (0.86)	T8 (0.96)	Ilium left (0.63)		
8	Baseline	left (0.94)	Rib (0.9)	Pelvis right (0.94)	L1 (0.99)	
9	Baseline	left (0.91)	Ilium right (0.96)			
10	Baseline	right (0.96)	L1 (0.73)			
11	Baseline	left (0.66)	L4 (0.89)			
12	Baseline	left (0.75)	T5 (0.97)	Sacrum right (0.99)	Ilium right (0.99)	Ilium left (0.97)
13	Baseline	right (0.91)	T8 (0.97)			
14	Baseline	right (1)	Iliac crest right (1)			
15	Baseline	right (0.92)	Sacrum left (0.98)	Sacrum left (0.99)		
16	Baseline	right (0.95)	T5 (0.98)			
17	Baseline	left (0.51)	Acetabulum left (0.93)			
18	Baseline	left (0.91)	Ilium right (0.93)			
19	Baseline	right (0.89)	Clavicle right (0.98)			
20	Baseline	right (0.96)	T12 (0.78)	T8 (0.95)	Acetabulum right (0.96)	
21	Baseline	left (0.89)	L3 (0.99)			
22	Baseline	left (0.77)	L5 (0.5)			
23	Baseline	left (0.74)	Sacrum right (1)			
24	Baseline	right (0.92)	T7 (0.72)			
25	Baseline	left (0.72)	T8 (0.89)	L2 (0.75)	L3 (0.78)	
26	Baseline	left (0.98)	Sacrum left (0.93)			
27	Baseline	left (0.89)	L1 (0.69)			
28	Baseline	left (1)	Ischium right (1)	Ilium right (1)		
28	Treated	right (0.6)	Pelvis left (0.81)			
29	Baseline	left (0.83)	Sacrum right (0.86)			
30	Baseline	left (0.93)	Anterior end of right second rib (0.99)			
31	Baseline	left (0.88)	Ilium left (0.94)	T10 (0.96)	L1 (0.93)	
32	Baseline	right (0.58)	Sacrum left (0.9)			
32	Treated	right (0.82)	Sacrum right (0.63)			
33	Baseline	right (0.52)	Sacrum right (0.88)			
34	Baseline	right (0.77)	Pleural fluid (0.99)			
35	Baseline	left (0.94)	Posterior iliac wing right (0.95)	Upper sacrum right (0.95)		
36	Baseline	right (0.8)	Iliac wing left (0.94)			

PID	Time point	Posterior iliac crest	FNAS 1	FNAS 2	FNAS 3	FNAS 4
37	Baseline	left (0.94)	Anterior ilium left (0.94)			
38	Baseline	right (1)	Sacrum right (1)			
39	Baseline	right (0.95)	Sacrum right (0.96)			
40	Baseline	right (0.6)	Ilium right (0.92)			
41	Baseline	left (0.84)	T8 (0.91)			
42	Baseline	left (0.93)	Posterior ilium right (0.85)			
43	Treated	right (0.68)	Sacrum right (0.76)	T9 (0.72)		
44	Treated	right (0.48)	Sacrum right (0.98)			
45	Treated	-	T3 (0.77)	T3 (0.89)		
46	Treated	left (0.48)	L4 (0.52)			
47	Treated	left (0.58)	Sacrum right (0.95)			
48	Treated	left (0.77)	Ischium left (0.93)	Ilium left (0.99)	T8 (0.95)	
49	Treated	left (0.9)	Right chest wall mass (0.98)			
50	Treated	right (0.87)	L2 (0.85)	Ilium left (0.93)		
51	Treated	right (0.89)	T12 (0.88)			

Values in brackets correspond to sample purity predicted based on WES data; FNAS: fine needle aspirate
