# Supplementary material for: Spatial genomic heterogeneity in multiple myeloma revealed by multi-region sequencing
Source: Nat Commun. 2017 Aug 16;8:268. doi: 10.1038/s41467-017-00296-y (PMC5559527; doi:10.1038/s41467-017-00296-y)
Supplement: Supplementary file 3 — Supplementary Data 2 [file 41467_2017_296_MOESM3_ESM.pdf]

Supplementary Data 2: Shared and unshared copy number aberrations.

PID	Time point	RNAS	FNAS	Type	Region	Copy RNAS	Copy FNAS
**1**	**Baseline**	**Iliac crest left**	**L4**	shared	5p15.33-5q11.1	3	3
				shared	7	3	3
				shared	9	3	3
				shared	15q11.1-15q11.2	3	3
				shared	15q14	3	3
				shared	19p13.3-19q12	3	3
				unshared	1p36.33-1p36.31	2	1
				unshared	1p36.12	2	1
				unshared	1p36.11	2	1
				unshared	1p36.11-1p35.1	2	1
				unshared	1p34.3-1p11.2	2	1
				unshared	3p21.1	2	3
				unshared	4	2	1
				unshared	5q11.2-5q21.1	3	1
				unshared	5q21.1-5q21.2	3	2
				unshared	5q21.3-5q35.3	3	1
				unshared	13	2	1
				unshared	15q11.2-15q13.3	3	2
				unshared	15q13.3-15q14	3	2
				unshared	15q14-15q26.3	3	2
				unshared	16p13.3-16p12.1	2	1
				unshared	17p13.3-17q21.31	2	1
				unshared	17q21.31-17q25.3	2	2 LOH
				unshared	19q12-19q13.12	3	2
				unshared	19q13.12-19q13.43	3	1
**2**	**Baseline**	**Iliac crest left**	**Ilium right**	shared (minor sub-clone in RNAS)	1q21.1-1q44	2 LOH	2 LOH
				shared	3	3	3
				shared	7	3	3
				shared	8p23.3-8p11.1	1	1
				shared	9	4	4
				shared	11	3	3
				shared	15	4	4
				shared	19p13.3-19q13.43	3	3
				unshared	4p15.1-4p16.3	2 LOH	1
				unshared	4p15.1-4q35.2	2 LOH	3
				unshared	5	4	3
				unshared	6q	2	1
				unshared	8q	3	2
**3**	**Baseline**	**Iliac crest right**	**Symphysis pubis**	shared	11q13.3-11q25	3	3
				unshared	-	-	-

PID	Time point	RNAS	FNAS	Type	Region	Copy RNAS	Copy FNAS
**4**	**Baseline**	**Iliac crest right**	**Ilium left**	shared	8p23.3-8p11.1	1	1
				shared	8q11.23-8q13.3	1	1
				shared	8q21.13-8q24.3	1	1
				shared	9p24.3-9q34.3	3	3
				shared	12q11-12q12	1	1
				shared	12q12-12q24.33	1	1
				shared	14q22.3-14q32.33	1	1
				shared	16p11.2-16p11.1	3	3
				shared	17p13.3-17p13.1	3	3
				shared	17p13.1	1	1
				shared	17p13.1-17p12	3	3
				shared	17p12	1	1
				shared	17p12-17p11.1	3	3
				shared	17q11.1-17q11.2	1	1
				shared	17q12-17q21.31	1	1
				shared	17q21.31-17q21.32	3	3
				shared	17q21.32-17q21.33	1	1
				shared	17q21.33-17q25.3	3	3
				shared	20p12.3-20p12.2	1	1
				shared	22q11.1-22q12.2	1	1
				shared	22q12.3-22q13.1	4	4
				unshared	1q	2	2 LOH
				unshared	16q11.2-16q23.3	2	1
				unshared	16q23.3-16q24.3	2	2 LOH
				unshared	22q12.2-22q12.3	3	1
				unshared	22q12.3	3	4
				unshared	22q13.1-22q13.2	1	2
**5**	**Baseline**	**Iliac crest right**	**Ilium right**	shared (minor sub-clone in RNAS)	1q21.1-1q44	4	4
				shared	4p16.3	1	1
				shared	6p11.2-6q11.1	3	3
				shared	8p23.3-8p11.21	1	1
				shared (minor sub-clone in RNAS)	11	3	3
				shared	12p11.1-12q11	3	3
				shared	16p11.2-16q24.3	3	3
				unshared	2	2	3
				unshared	3p26.3-3p14.1	2	2 LOH
				unshared	3p14.1-3q29	2	3
				unshared	4p16.2-4q35.2	2	3
				unshared	6p25.3-6q11	2	3

PID	Time point	RNAS	FNAS	Type	Region	Copy RNAS	Copy FNAS
				unshared	6q12	3	5
				unshared	6q12-6q27	1	2 LOH
				unshared	7	2	3
				unshared	8p11.21-8q24.3	3	4
				unshared	9	2	3
				unshared	15q22.31-15q26.3	2	3
				unshared	16p13.3-16p11.2	2	3
				unshared	19	2	3
				unshared	20	2	3
				unshared	22	2	3
**6**	**Baseline**	**Iliac crest left**	**Sacrum right**	shared	3	3	3
				shared	4	3	3
				shared	5	4	4
				shared	6	3	3
				shared	9p24.3-9p21.1	4	4
				shared	9p21.1-9p11.2	3	3
				shared	9q12-9q33.2	4	4
				shared	9q33.2-9q34.11	3	3
				shared	9q34.11-9q34.3	4	4
				shared	11	3	3
				shared	15	4	4
				shared	19	3	3
				shared	20	3	3
				shared	21	3	3
				unshared	-	-	-
**7**	**Baseline**	**Iliac crest right**	**T8**	shared	2p25.3-2p11.2	3	3
				shared	5	3	3
				shared	6p25.3-6q23.3	3	3
				shared	8q12.3-8q24.21	3	3
				shared	9	3	3
				shared	10p15.3-10p13	1	1
				shared	10p12.1-10p11.22	1	1
				shared	10p11.22-10q11.21	1	1
				shared	11	3	3
				shared	15	5	5
				shared	19p13.3-19q13.43	3	3
				shared	21	3	3
				unshared	1q41	1	2
				unshared	1q41-1q42.12	1	2
		**Iliac crest right**	**Ilium left**	shared	2p25.3-2p13.1	3	3
				shared	5	3	3
				shared	6p25.3-6q22.31	3	3

PID	Time point	RNAS	FNAS	Type	Region	Copy RNAS	Copy FNAS
				shared	9	3	3
				shared	10p15.3-10p13	1	1
				shared	10p12.1-10p11.22	1	1
				shared	10p11.22-10q11.21	1	1
				shared	11p14.1-11q25	3	3
				shared	15	5	5
				shared	19p13.3-19q13.42	3	3
				shared	21	3	3
				unshared	1q41	1	2
				unshared	1q41-1q42.12	1	2
				unshared	2p13.1-2p11.2	3	3
				unshared	4q13.2-4q35.2	2	3
				unshared	6q22.31-6q23.3	3	2 LOH
				unshared	6q23.3-6q27	2	2 LOH
				unshared	8q12.3-8q24.21	3	2
				unshared	11p15.5-11p14.1	3	2
				unshared	19q13.42-19q13.43	3	2
**8**	**Baseline**	**Iliac crest left**	**Rib**	shared	6p25.3-6q22.32	3	3
				shared	6q22.32-6q22.33	1	1
				shared	6q22.33	3	3
				shared	6q23.1-6q23.2	1	1
				shared	6q23.2-6q23.3	3	3
				shared	6q23.3-6q24.1	1	1
				shared	6q24.1	3	3
				shared	6q24.1-6q27	1	1
				shared	7	3	3
				shared	8q24.21	3	3
				shared	13	1	1
				shared	15	3	3
				shared	19	3	3
				shared	22q11.21-22q11.23	3	3
				unshared	1q	4	4 LOH
		**Iliac crest left**	**Pelvis right**	shared	**like rib**		
				additional shared	1q21.1-1q44	4	4
				unshared	-	-	-
		**Iliac crest left**	**L1**	shared	**like pelvis right**		
				unshared	-	-	-
**9**	**Baseline**	**Iliac crest left**	**Ilium right**	shared	16q11.2-16q24.3	2 LOH	2 LOH
				unshared	-	-	-
**10**	**Baseline**	**Iliac crest right**	**L1**	shared	1q21.1-1q44	3	3
				shared	2q37.3-2q37.3	1	1

PID	Time point	RNAS	FNAS	Type	Region	Copy RNAS	Copy FNAS
				shared	6q22.31-6q27	1	1
				shared	9p13.2-9p13.1	4	4
				shared	9q21.33-9q22.31	1	1
				shared	11p15.5-11p11.12	1	1
				shared	11q13.1-11q13.4	4	4
				shared	11q13.4-11q14.1	3	3
				shared	11q14.2-11q22.2	1	1
				shared	11q22.3-11q23.1	3	3
				shared	11q23.1-11q23.3	1	1
				shared	11q23.3	3	3
				shared	11q23.3-11q24.1	4	4
				shared	11q24.1-11q24.2	1	1
				shared	11q24.2-11q25	3	3
				shared	13	1	1
				shared	16q12.1-16q24.3	1	1
				unshared	4p16.3	1	2
				unshared	5p15.33-5p15.2	3	2
				unshared	15q21.1-15q26.3	3	2
**11**	**Baseline**	**Iliac crest left**	**L4**	shared	3	3	3
				shared	5	3	3
				shared	6p25.3-6p11.1	3	3
				shared	6q11.1-6q27	2 LOH	2 LOH
				shared	7	3	3
				shared	8p23.3-8p11.21	1	1
				shared	9	3	3
				shared	13	1	1
				shared	14q22.1-14q31.1	1	1
				shared	15	3	3
				shared	19	3	3
				shared	20p13-20p11.21	1	1
				shared	21	3	3
				unshared	-	-	-
**12**	**Baseline**	**Iliac crest left**	**T5**	shared	5	3	3
				shared	9p24.3-9p13.2	3	3
				shared	9p13.2-9p11.2	3	3
				shared	15q11.1-15q22.31	3	3
				shared	15q22.33-15q26.3	3	3
				shared	19p13.3-19p11	3	3
				unshared	1p36.33-1p13.1	2	2 LOH
				unshared	1p32.3	2	0
				unshared	1q	2	3
				unshared	3q26.32	2	1
				unshared	4q13.1-4q22.1	2	1
				unshared	7p12.3	1	2

PID	Time point	RNAS	FNAS	Type	Region	Copy RNAS	Copy FNAS
				unshared	8p	2	1
				unshared	9p13.2	3	2 LOH
				unshared	9q	3	2 LOH
				unshared	15q22.31-15q22.33	3	2
				unshared	17p13.3-17p12	2	1
				unshared	19q	3	2
				unshared	22q11.21	2	3
				unshared	22q11.23-22q13.33	2	1
		**Iliac crest left**	**Sacrum right**	shared	**Like T5**		
				unshared	**Like T5**		
		**Iliac crest left**	**Ilium right**	shared	**Like T5**		
				unshared	**Like T5**		
		**Iliac crest left**	**Ilium left**	shared	**Like T5**		
				unshared	**Like T5**		
				additional unshared	3q26.33	2	1
**13**	**Baseline**	**Iliac crest right**	**T8**	shared	1q24.2-1q25.2	1	1
				shared	3p26.3-3p21.31	3	3
				shared	3p21.31-3q13.31	3	3
				shared	3q13.31-3q13.33	2 LOH	2 LOH
				shared	3q13.33-3q22.1	1	1
				shared	3q22.1-3q23	2 LOH	2 LOH
				shared	3q23	1	1
				shared	3q23-3q24	2 LOH	2 LOH
				shared	3q24-3q29	3	3
				shared	5p15.33-5q22.1	3	3
				shared	5q22.3-5q31.1	3	3
				shared	5q34-5q35.3	3	3
				shared	8q24.21-8q24.3	1	1
				shared	9q21.11-9q22.33	3	3
				shared	10q24.32-10q25.2	1	1
				shared	11p15.5-11p13	3	3
				shared	11p13-11p12	2 LOH	2 LOH
				shared	11p12-11q13.1	3	3
				shared	11q13.2-11q22.3	2 LOH	2 LOH
				shared	11q22.3-11q23.1	4	4
				shared	11q23.1-11q23.3	2 LOH	2 LOH
				shared	11q23.3	3	3
				shared	11q23.3-11q24.3	4	4
				shared	11q24.3-11q25	5	5
				shared	11q25	3	3
				shared	11q25	2 LOH	2 LOH

PID	Time point	RNAS	FNAS	Type	Region	Copy RNAS	Copy FNAS
				shared	12p13.31-12p12.2	1	1
				shared	12p11.1-12q12	3	3
				shared	15	3	3
				shared	16p13.3	1	1
				shared	16q11.2-16q22.1	3	3
				shared	16q23.3-16q24.1	1	1
				shared	19p13.3-19q13.11	3	3
				shared (minor sub-clone at T8)	19q13.11-19q13.43	3	3
				shared	19q13.43	3	3
				shared	21p11.2-21q11.2	3	3
				shared	22q11.1-22q11.21	1	1
				unshared	1p31.3-1p21.1	2	1
				unshared	1p21.1-1p13.3	2	1
				unshared	1p13.2-1p12	2	1
				unshared	1q25.2-q25.3	2	1
				unshared	2p22.3-p22.1	2	1
				unshared	4q11-4q12	2	1
				unshared	4q21.1-4q24	2	1
				unshared	4q26-4q27	2	1
				unshared	4q28.3-4q31.1	2	1
				unshared	4q31.21-4q31.23	2	1
				unshared	5q22.1-4q22.3	3	2
				unshared	5q31.1-4q31.3	3	2 LOH
				unshared	5q31.3-5q32	3	1
				unshared	5q32-5q33.3	2	1
				unshared	5q33.3-5q34	2	2 LOH
				unshared	5q34	2	3
				unshared	6q21	2	1
				unshared	6q24.1-6q24.2	1	2
				unshared	6q25.2-6q25.3	1	2
				unshared	8p21.2-8p21.1	2	1
				unshared	8p11.21-8p11.1	2	1
				unshared	9	4	3
				unshared	12p13.33-12p13.31	1	2
				unshared	12p12.2-12p11.1	1	2
				unshared	12q21.31-q23.3	1	2
				unshared	13q13.3-q21.31	1	2
				unshared	14q21.1	2	1
				unshared	18	3	2 LOH
**14**	**Baseline**	**Iliac crest right**	**Iliac crest right**	shared	11q13.3-11q25	3	3
				unshared	7p22.3-7p21.2	2	1

PID	Time point	RNAS	FNAS	Type	Region	Copy RNAS	Copy FNAS
**15**	**Baseline**	**Iliac crest right**	**Sacrum left FL1**	shared	1p22.3-1p11.2	1	1
				shared	1q25.1-1q25.3	1	1
				shared (minor sub-clone in RNAS)	2p25.3-2p23.2	1	1
				shared	3	3	3
				shared	4q31.22-4q31.23	1	1
				shared	5p15.33-5q32	3	3
				shared	5q33.1	2 LOH	2 LOH
				shared	5q33.1-5q33.3	3	3
				shared	6q14.1-6q27	1	1
				shared	8p23.3-8p11.22	1	1
				shared	9	3	3
				shared	11p11.2-11q25	3	3
				shared	12p13.33-12p12.3	1	1
				shared	12p11.21-12q13.11	1	1
				shared	13	1	1
				shared	15	4	4
				shared	19p13.3-19q13.2	3	3
				shared	19q13.31-19q13.43	3	3
				shared	22q11.1-22q11.21	1	1
				shared	22q12.1-22q12.2	1	1
				shared	22q12.3	1	1
				shared	22q13.31-22q13.33	1	1
				unshared	12q21.1-12q24.12	2	1
		**Iliac crest right**	**Sacrum left FL 2**	shared	**like Sacrum left FL1**		
				unshared	**like Sacrum left FL1**		
**16**	**Baseline**	**Iliac crest right**	**T5**	shared	1p31.1-1p11.2	1	1
				shared	1q21.1-1q21.3	3	3
				shared	1q24.2-1q24.3	3	3
				shared	1q31.2-1q43	3	3
				shared	4p16.3-4q12	1	1
				shared	4q12-4q13.1	1	1
				shared	4q13.1-4q13.3	1	1
				shared	4q28.2-4q35.2	1	1
				shared	8q23.1-8q24.3	3	3
				shared	10p15.3-10p15.1	1	1
				shared	12p13.33-12p12.3	3	3
				shared	13q11-13q14.21.1	1	1
				shared	13q32.3-13q34	1	1
				shared	15q13.1-15q21.1	3	3

PID	Time point	RNAS	FNAS	Type	Region	Copy RNAS	Copy FNAS
				shared	15q21.1-15q26.3	4	4
				shared	16p13.3-16q24.3	1	1
				shared	17p13.3-17p12	1	1
				shared	17q23.2-17q25.3	1	1
				unshared	5p13.1-15.33	4	3
				unshared	12q24.31-12q24.32	2	1
**17**	**Baseline**	**Iliac crest left**	**Acetabulum left**	shared	1q21.1-1q44	3	3
				shared	3	3	3
				shared	5	3	3
				shared	7	3	3
				shared	15	3	3
				shared	16q11.2-16q24.3	2 LOH	2 LOH
				shared	19	3	3
				unshared	-	-	-
**18**	**Baseline**	**Iliac crest left**	**Ilium right**	shared	2	3	3
				shared	3	3	3
				shared	4	3	3
				shared	5	3	3
				shared	7	3	3
				shared	9	3	3
				shared	11p15.5-11p11.12	3	3
				shared	11p11.12-11q25	4	4
				shared	15	5	5
				shared	19	4	4
				shared	20	3	3
				shared	21	3	3
				unshared	-	-	-
**19**	**Baseline**	**Iliac crest right**	**Clavicle right**	shared	1q21.1-1q44	3	3
				shared	2p11.2	1	1
				shared	3	3	3
				shared	5p15.33-5q11.2	3	3
				shared	6p25.3-6p11.2	3	3
				shared	6q13-6q27	3	3
				shared	7p22.3-7q36.3	3	3
				shared	8p23.3-8p12	1	1
				shared	9	3	3
				shared	11	3	3
				shared	12p13.33-12p11.1	1	1
				shared	13	1	1
				shared	14q11.2-14q32.33	2 LOH	2 LOH
				shared	15	3	3
				shared	18p11.32-18q23	3	3

PID	Time point	RNAS	FNAS	Type	Region	Copy RNAS	Copy FNAS
				shared	19	3	3
				shared	20p12.1-20q11.21	3	3
				shared	21	2 LOH	2 LOH
				shared	22	2 LOH	2 LOH
				unshared	5q	3	2 LOH
				unshared	20p13-20p12.1	3	2
**20**	**Baseline**	**Iliac crest right**	**T12**	shared	3	3	3
				shared	4p14-4q35.2	3	3
				shared	5	3	3
				shared	6p25.3-6q14.1	3	3
				shared	6q14.2-6q16.1	3	3
				shared	6q16.1	4	4
				shared	6q16.1	3	3
				shared	6q16.3-6q21	3	3
				shared	6q21-6q22.31	4	4
				shared	6q22.31	3	3
				shared	7p22.3-7q11.21	3	3
				shared	7q11.21-7q36.3	3	3
				shared	9	3	3
				shared	11q12.1-11q25	3	3
				shared	15q11.1-15q23	3	3
				shared	15q23-15q24.3	3	3
				shared	15q25.3-15q26.3	3	3
				shared	17p11.2-17q25.3	3	3
				shared	19p13.3-19p13.12	3	3
				shared	19p13.12-19p12	4	4
				shared	19p12-19q13.43	3	3
				shared (minor sub-clone in RNAS)	21	3	3
				shared	22q11.21-22q13.1	1	1
				shared	22q13.1	3	3
				shared	22q13.2-22q13.33	1	1
				unshared	15q23	4	3
				unshared	15q24.3-15q25.3	4	3
				unshared	17p13.3-17p11.2	3	2
		**Iliac crest right**	**T8**	shared	**Like T12**		
				additional shared	15q23	4	4
				additional shared	15q24.3-15q25.3	4	4
				additional shared	17p13.3-17p11.2	3	3

PID	Time point	RNAS	FNAS	Type	Region	Copy RNAS	Copy FNAS
				unshared	-	-	-
		**Iliac crest right**	**Acetabulum right**	shared	**Like T12**		
				unshared	**Like T12**		
**21**	**Baseline**	**Iliac crest left**	**L3**	shared	1p36.33-1p22.3	3	3
				shared	1p22.3-1p12	1	1
				shared	1q21.1-1q32.3	3	3
				shared	1q42.2-1q42.3	3	3
				shared	2p25.3-2p23.3	1	1
				shared	2p23.3-2p22.2	3	3
				shared	2p16.3	3	3
				shared	8p21.1-8p11.22	1	1
				shared	11q13.1-11q13.2	3	3
				shared	11q13.3-11q25	3	3
				shared	13	1	1
				shared	14q32.33	1	1
				shared	17q12	3	3
				shared	22	1	1
				unshared	-	-	-
**22**	**Baseline**	**Iliac crest left**	**L5**	shared	1p36.33-1p11.2	2 LOH	2 LOH
				shared	9q12-9q34.3	2 LOH	2 LOH
				shared	13	1	1
				unshared	-	-	-
**23**	**Baseline**	**Iliac crest left**	**Sacrum right**	shared	3p11.1-3q29	3	3
				shared	6p25.3-6p27	4	4
				shared	9p24.3-9q34.3	3	3
				shared	11p15.5-11q25	3	3
				shared	15q11.1-15q26.3	3	3
				shared	19p13.3-19p11	3	3
				shared	20q11.22	1	1
				shared	21p11.2-21q22.3	3	3
				shared	22q11.1	3	3
				unshared	-	-	-
**24**	**Baseline**	**Iliac crest right**	**T7**	shared	3p22.3-3p22.2	1	1
				shared	3p22.2-3q29	3	3
				shared	5	3	3
				shared	6p24.3-6p12.3	4	4
				shared	6p12.3-6q22.32	3	3
				shared	6q22.32-6q27	2 LOH	2 LOH
				shared	7	3	3
				shared	8p22-8p11.21	1	1

PID	Time point	RNAS	FNAS	Type	Region	Copy RNAS	Copy FNAS
				shared	8q24.21-8q24.3	3	3
				shared	9p24.3-9p11.2	4	4
				shared	9q12-9q34.3	3	3
				shared	13	1	1
				shared	15	4	4
				shared	19	3	3
				shared	21	3	3
				unshared	1q	3	4
**25**	**Baseline**	**Iliac crest left**	**T8**	shared	5	3	3
				shared	6p25.3-6q16.2	3	3
				shared	7	3	3
				shared	9	3	3
				shared	15	3	3
				shared	19	3	3
				shared	21q22.12-21q22.3	1	1
				unshared	-	-	-
		**Iliac crest left**	**L2**	shared	**Like T8**		
				unshared	-	-	-
		**Iliac crest left**	**L3**	shared	**Like T8**		
				unshared	-	-	-
**26**	**Baseline**	**Iliac crest left**	**Sacrum left**	shared	5	3	3
				shared	6p25.3-6p21.1	3	3
				shared	6p21.1-6q27	1	1
				shared	11	3	3
				shared	13	1	1
				shared	14q11.2-14q32.33	2 LOH	2 LOH
				shared	14q32.33-14q32.33	0	0
				shared	15	3	3
				shared	19	3	3
				shared	21	3	3
				unshared	-	-	-
**27**	**Baseline**	**Iliac crest left**	**L1**	shared	1q21.1-1q32.2	3	3
				shared	1q43-1q44	3	3
				shared	3	3	3
				shared	5	3	3
				shared	7	3	3
				shared	8q24.13-8q24.21	1	1
				shared	9	3	3
				shared	14	3	3
				shared	15	3	3
				shared	19p13.3-19p13.11	4	4

PID	Time point	RNAS	FNAS	Type	Region	Copy RNAS	Copy FNAS
				shared	19p13.11-19p13.11	3	3
				unshared	-	-	-
**28**	**Baseline**	**Iliac crest left**	**Ischium right**	shared	1p36.33-1p11.2	3	3
				shared	1q21.1-1q44	5	5
				shared	2	4	4
				shared	3	5	5
				shared	4	4	4
				shared	5	5	5
				shared	6p25.3-6q24.3	5	5
				shared	6q24.3-6q27	3	3
				shared	7	5	5
				shared	8	4	4
				shared	9p24.3-9p11.2	4	4
				shared	10	4	4
				shared	11p15.5-11p15.2	5	5
				shared	11p15.2-11p11.2	4	4
				shared	11p11.2-11q12.1	5	5
				shared	11q12.1	4	4
				shared	11q12.1-11q14.1	5	5
				shared	11q14.1-11q22.3	4	4
				shared	11q22.3-11q25	5	5
				shared	12	3	3
				shared	13	3	3
				shared	14q11.2-14q24.1	5	5
				shared	14q24.1-14q32.13	4 LOH	4 LOH
				shared	14q32.13-14q32.33	5	5
				shared	14q32.33	4 LOH	4 LOH
				shared	14q32.33	5	5
				shared	15q11.1-15q15.1	6	6
				shared	15q15.1-15q22.31	5	5
				shared	15q22.31-15q24.1	6	6
				shared	15q24.1-15q24.2	5	5
				shared	15q24.2-15q25.2	6	6
				shared	15q25.2-15q26.3	5	5
				shared	16	3	3
				shared	17p13.3-17p13.1	3	3
				shared	17q21.31-17q25.3	4	4
				shared	18	4	4
				shared	19p13.3-19p13.11	6	6
				shared	19p13.11-19q13.43	5	5
				shared	20	4	4
				shared	21	6	6
				shared	22	3	3
				unshared	9q13-9q31.1	5	4

PID	Time point	RNAS	FNAS	Type	Region	Copy RNAS	Copy FNAS
		**Iliac crest left**	**Ilium right**	shared	**Like Ischium right**		
				unshared	**Like Ischium right**		
	**Treated**	**Iliac crest right**	**Pelvis left**	shared	1q21.1-1q44	3	3
				shared	3	3	3
				shared	5	3	3
				shared	6p25.3-6q24.3	3	3
				shared	7	3 LOH	3 LOH
				shared	8	3	3
				shared	9p24.3-9q31.1	3	3
				shared	9q31.1-9q34.3	3 LOH	3 LOH
				shared	10q11.21-10q11.22	3	3
				shared	11p15.5-11p15.2	3	3
				shared	11p12-11q12.1	3	3
				shared	11q12.1-11q14.1	3	3
				shared	11q22.3-11q25	3	3
				shared	13	1	1
				shared	14q11.2-14q24.1	3	3
				shared	14q24.1-14q32.13	2 LOH	2 LOH
				shared	14q32.13-14q32.33	3	3
				shared	14q32.33	2 LOH	2 LOH
				shared	14q32.33	3	3
				shared	15q11.1-15q15.1	4	4
				shared	15q15.1-15q22.31	3	3
				shared	15q22.31-15q24.1	4	4
				shared	15q24.1-15q24.2	3	3
				shared	15q24.2-15q25.2	4	4
				shared	15q25.2-15q26.3	3	3
				shared	17p13.3-17p13.1	3	3
				shared	17q11.2-17q21.31	1	1
				shared	17q21.31-17q25.3	3	3
				shared	19p13.3-19p13.11	4	4
				unshared	-	-	-
**29**	**Baseline**	**Iliac crest left**	**Sacrum right**	shared	1q21.1-1q44	3	3
				shared	5q14.2-5q33.2	1	1
				shared	9p24.3-9p23	1	1
				shared	11p15.5-11p14.3	2 LOH	2 LOH
				shared	11p14.3-11p12	1	1
				shared	13q11-13q31.1	1	1
				shared	13q31.2-13q32.2	1	1
				unshared	-	-	-

PID	Time point	RNAS	FNAS	Type	Region	Copy RNAS	Copy FNAS
**30**	**Baseline**	**Iliac crest left**	**Anterior end of right second rib**	shared	1p33-1p31.3	1	1
				shared	1p31.1-1p31.1	1	1
				shared	1p31.1-1p12	1	1
				shared	1q12-1q44	3	3
				shared	3	3	3
				shared	6q24.3-6q27	1	1
				shared	7	3	3
				shared	13	1	1
				shared	15	3	3
				shared	16p11.2-16q24.3	1	1
				shared	20q13.2-20q13.2	1	1
				shared	21	4	4
				unshared	8	2	3
**31**	**Baseline**	**Iliac crest left**	**Ilium left**	shared	1p36.11-1p35.3	1	1
				shared	1p35.2-1p35.1	1	1
				shared	1p33-1p32.1	1	1
				shared	1p31.1-1p12	1	1
				shared	2q24.1-2q24.3	1	1
				shared	3p26.3-3p12.2	1	1
				shared	3p12.1-3p11.1	1	1
				shared	4q34.3	1	1
				shared	4q35.2	3	3
				shared	6q22.33-6q27	1	1
				shared	7	3	3
				shared	12q13.11-12q13.12	1	1
				shared	12q13.13-12q13.3	1	1
				shared	12q14.1-12q24.33	1	1
				shared	15	3	3
				shared	17p12	1	1
				shared	19p13.3-19q13.43	3	3
				unshared	-	-	-
		**Iliac crest left**	**T10**	shared	**Like ilium left**		
				unshared	-	-	-
		**Iliac crest left**	**L1**	shared	**Like ilium left**		
				unshared	-	-	-
**32**	**Baseline**	**Iliac crest right**	**Sacrum left**	shared	1q21.1-1q44	3	3
				shared	8p23.3-8p22	1	1
				shared	8p21.3-8p12	1	1
				shared	8p12	1	1
				shared	11q13.3-11q13.4	3	3

PID	Time point	RNAS	FNAS	Type	Region	Copy RNAS	Copy FNAS
				shared	13q12.13-13q21.32	1	1
				shared	15q26.2-15q26.3	1	1
				shared	16q11.2-16q24.3	1	1
				shared	17q21.31-17q25.3	3	3
				unshared	-	-	-
	**Treated**	**Iliac crest right**	**Sacrum right**	shared	**Like at baseline**		
				unshared	-		
**33**	**Baseline**	**Iliac crest right**	**Sacrum right**	shared	2p22.3	3	3
				shared	3p26.3-3p14.3	3	3
				shared	3p14.3	4	4
				shared	3p14.3-3q29	3	3
				shared	4	2 LOH	2 LOH
				shared	5	3	3
				shared	9	3	3
				shared	11p15.5-11q14.2	3	3
				shared	11q14.2-11q25	3	3
				shared	13	1	1
				shared	15q11.1-15q21.2	4	4
				shared	15q21.3-15q26.3	4	4
				shared	16p12.2-16p11.2	3	3
				shared	17q23.3-17q25.3	1	1
				shared	19p13.3-19q13.2	4	4
				shared	19q13.2-19q13.43	3	3
				shared	20q11.21-20q13.12	1	1
				unshared	-	-	-
**34**	**Baseline**	**Iliac crest right**	**Pleural fluid**	shared	1p32.3-1p11.2	1	1
				shared	1q21.1-1q23.1	4	4
				shared	1q23.1-1q44	3	3
				shared	2q23.3	1	1
				shared	2q24.3	1	1
				shared	3p26.3-3p14.2	1	1
				shared	4p16.3	1	1
				shared	6q25.1-6q26	1	1
				shared	7p22.3-7p14.3	3	3
				shared	7q11.21-7q36.3	3	3
				shared	10q11.22	3	3
				shared	11	2 LOH	2 LOH
				shared	13	1	1
				shared	14	1	1
				shared	17p13.2-17p12	1	1
				shared	19p13.3	2 LOH	2 LOH

PID	Time point	RNAS	FNAS	Type	Region	Copy RNAS	Copy FNAS
				shared	19p13.3-19p13.2	3	3
				shared	20p13-20p11.1	1	1
				shared	21p11.2-21q22.3	3	3
				shared	22q12.2-22q13.33	1	1
				unshared	-	-	-
**35**	**Baseline**	**Iliac crest left**	**Posterior iliac wing right**	shared	3	3	3
				shared	5	4	4
				shared	7p22.3-7q21.3	3	3
				shared	7q21.3-7q36.3	4	4
				shared	8p23.3-8p11.21	1	1
				shared	9	4	4
				shared	11	3	3
				shared	14q24.2-14q32.33	1	1
				shared	15	4	4
				shared	19	3	3
				shared	21	3	3
				unshared	-	-	-
		**Iliac crest left**	**Upper sacrum right**	shared	**Like posterior iliac wing right**		
				unshared	-	-	-
**36**	**Baseline**	**Iliac crest right**	**Iliac wing left**	shared	8p23.3-8p12	1	1
				shared	15q11.1-15q11.2	2 LOH	2 LOH
				shared	17q21.31-17q25.3	3	3
				unshared	-	-	-
**37**	**Baseline**	**Iliac crest left**	**Anterior ilium left**	shared	5	3	3
				shared	9	3	3
				shared	11	3	3
				shared	15	3	3
				shared	19	3	3
				unshared	-	-	-
**38**	**Baseline**	**Iliac crest right**	**Sacrum right**	shared	3p14.2	3	3
				shared	9p13.3-9p13.2	3	3
				shared	9q22.32-9q22.33	1	1
				shared	9q33.3-9q34.11	1	1
				shared	11q13.1	3	3
				shared	13q11-13q12.12	1	1
				shared	13q14.13	1	1
				shared	13q32.1-13q32.2	1	1
				shared	19p13.2-19p13.12	3	3

PID	Time point	RNAS	FNAS	Type	Region	Copy RNAS	Copy FNAS
				shared	22q11.1-22q11.21	3	3
				unshared	-	-	-
**39**	**Baseline**	**Iliac crest right**	**Sacrum right**	shared	1p31.1-1p13.1	1	1
				shared	7q31.1-7q36.3	3	3
				shared	8p23.3-8p21.2	1	1
				shared	11q13.3-11q13.5	3	3
				shared	11q13.5-11q22.1	1	1
				shared	11q22.1-11q22.2	0	0
				shared	11q22.3-11q23.2	1	1
				shared	11q23.2-11q23.3	0	0
				shared	11q23.3	1	1
				shared	11q23.3	3	3
				shared	11q23.3-11q24.1	1	1
				shared	11q24.1-11q25	3	3
				shared	12p13.33-12p12.3	1	1
				shared	13q14.12-13q21.33	1	1
				shared	14q31.3-14q32.33	3	3
				shared	16	1	1
				shared	20p13-20p12.1	1	1
				shared	22	1	1
				unshared	-	-	-
**40**	**Baseline**	**Iliac crest right**	**Ilium right**	shared	1p36.33-1p11.2	1	1
				shared	2	1	1
				shared	4	1	1
				shared	13	1	1
				shared	22	1	1
				unshared	-	-	-
**41**	**Baseline**	**Iliac crest left**	**T8**	shared	3	3	3
				shared	5	3	3
				shared	8	2 LOH	2 LOH
				shared	9	4	4
				shared	11	3	3
				shared	12q24.22-12q24.33	2 LOH	2 LOH
				shared	15	3	3
				shared	18	2 LOH	2 LOH
				shared	19	3	3
				unshared	-	-	-
**42**	**Baseline**	**Iliac crest left**	**Posterior ilium right**	shared	1p36.33-1p11.2	2 LOH	2 LOH
				shared	1q21.1-1q44	3 LOH	3 LOH
				shared	2	3	3

PID	Time point	RNAS	FNAS	Type	Region	Copy RNAS	Copy FNAS
				shared	3	3	3
				shared	4	3	3
				shared	5p15.33-5p14.3	3	3
				shared	5p14.3-5p13.3	4	4
				shared	5p13.3	3	3
				shared	5p13.3-5p13.2	4	4
				shared	5p13.2-5p13.1	5	5
				shared	5p13.1-5q35.3	4	4
				shared	6	3	3
				shared	7	3	3
				shared	8	3	3
				shared	9	3	3
				shared	10	3	3
				shared	11p15.5-11q23.3	3	3
				shared	11q23.3-11q25	4	4
				shared	15	4	4
				shared	16p13.3-16p11.1	2 LOH	2 LOH
				shared	16q11.2-16q24.3	1	1
				shared	17	3	3
				shared	19	4	4
				shared	20p13-20q11.21	4	4
				shared	20q11.21-20q13.33	3	3
				shared	21	3	3
				unshared	-	-	-
**43**	**Treated**	**Iliac crest right**	**Sacrum right**	shared	1p36.33-1p34.3	3	3
				shared	2p25.3-2q12.1	4	4
				shared	3p26.3-3q11.2	4	4
				shared	3q11.2-3q29	3	3
				shared	4p16.3-4q12	4	4
				shared	4q13.3-4q31.1	4	4
				shared	4q31.1-4q32.1	2 LOH	2 LOH
				shared	4q32.1-4q35.2	4	4
				shared	6p25.3-6q21	4	4
				shared	6q21-6q27	2 LOH	2 LOH
				shared	8p23.3-8p12	1	1
				shared	9p24.3-9q34.3	4	4
				shared	11q22.1-11q22.3	4	4
				shared	11q22.3-11q25	4	4
				shared	12	2 LOH	2 LOH
				shared	13	2 LOH	2 LOH
				shared	16p13.3-16p13.2	3	3
				shared	16q11.2-16q24.3	2 LOH	2 LOH
				shared	17q11.2-17q12	3	3
				shared	17q12-17q21.31	2 LOH	2 LOH

PID	Time point	RNAS	FNAS	Type	Region	Copy RNAS	Copy FNAS
				shared	18	4	4
				shared	21	4	4
				shared	22	2 LOH	2 LOH
				unshared	1p34.3-1q44	5	4
				unshared	2q12.1-2q34	4	3
				unshared	2q34-2q37.3	4	2
				unshared	4q12-4q13.2	4	3
				unshared	5	4	3
				unshared	7p	3 LOH	2 LOH
				unshared	7q	5	3
				unshared	8p12-8q24.3	4	3
				unshared	10	4	3
				unshared	11p13-11p15.5	4	3
				unshared	11q11-11q22.1	4	3
				unshared	14	4	3
				unshared	15	4	3
				unshared	16p11.1-16p13.2	4	3
				unshared	17p13.3-17q11.2	3	4
				unshared	17q21.31-17q25.2	5	4
				unshared	17q25.3	5	3
				unshared	19	5	4
				unshared	20p13-20p12.3	2 LOH	1
				unshared	20p12.3-20p11.21	2 LOH	3 LOH
				unshared	20p11.21-20q11.23	4	5
				unshared	20q11.23-20q13.33	4	3
		**Iliac crest right**	**T9**	shared	**Like sacrum right**		
				additional shared	19p13.11-19q13.43	5	5
				unshared	1p34.3-q44	5	4
				unshared	2q12.1-q34	4	3
				unshared	2q34-q37.3	4	2
				unshared	4q12-q13.2	4	3
				unshared	5	4	3
				unshared	7p	3 LOH	2 LOH
				unshared	7q	5	3
				unshared	8p12-q24.3	4	3
				unshared	10	4	3
				unshared	11p13-p15.5	4	3
				unshared	11q11-q22.1	4	3
				unshared	14	4	3
				unshared	15	4	3
				unshared	16p11.1-p13.2	4	3
				unshared	17p13.3-q11.2	3	4
				unshared	17q21.31-q25.2	5	4
				unshared	17q25.3	5	3

PID	Time point	RNAS	FNAS	Type	Region	Copy RNAS	Copy FNAS
				unshared	19p13.3-19p13.11	5	4
				unshared	20p13-20p12.3	2 LOH	1
				unshared	20p12.3	2 LOH	3 LOH
				unshared	20p12.3-20p12.1	2 LOH	4 LOH
				unshared	20p12.1-20p11.21	2 LOH	3 LOH
				unshared	20p11.21-q11.23	4	5
				unshared	20q11.23-q13.33	4	3
**44**	**Treated**	**Iliac crest right**	**Sacrum right**	shared	1p33-1p31.1	1	1
				shared	1p31.1-1p12	1	1
				shared	3p14.3-3q26.1	3	3
				shared	4q23-4q35.2	2 LOH	2 LOH
				shared	5p15.33-5q23.1	3	3
				shared	6q23.3-6q27	1	1
				shared	8p22	1	1
				shared	8p21.2-8p11.1	1	1
				shared	9	3	3
				shared	11p15.5-11p12	3	3
				shared	11p12-11p11.2	2 LOH	2 LOH
				shared	11p11.12-11q25	3	3
				shared	15q11.1-15q24.3	4	4
				shared	15q25.1-15q26.3	4	4
				shared	19p13.3-19q13.12	3	3
				unshared	3p26.3-3p14.3	3	2
				unshared	3q26.1-3q26.31	3	2
				unshared	4p16.3-4q23	2 LOH	1
				unshared	5q23.1-5q35.3	3	2
				unshared	10p12.2-10p12.1	2	1
				unshared	10q11.23-10q22.1	2	1
				unshared	10q22.3-10q23.1	2	1
				unshared	19q13.12-19q13.43	3	2
**45**	**Treated**	**T3**	**T3**	shared	1p36.33-1p13.2	3	3
				shared	1p13.2-1p12	2 LOH	2 LOH
				shared	1p12-1q44	3	3
				shared	2q36.1-2q37.3	1	1
				shared	3p26.3-3p13	3	3
				shared	3p13-3p12.3	3	3
				shared	3p12.3-3q29	3	3
				shared	4q13.1-4q22.3	1	1
				shared	5p15.33-5p15.31	5	5
				shared	5p15.31-5p15.2	3	3
				shared	5p15.2-5p11	5	5
				shared	5q11.1-5q11.2	3	3
				shared	5q11.2-5q35.3	2 LOH	2 LOH

PID	Time point	RNAS	FNAS	Type	Region	Copy RNAS	Copy FNAS
				shared	6q23.3-6q27	1	1
				shared	7p22.3-7p21.1	1	1
				shared	7p21.1-7q11.21	3	3
				shared	7q11.21-7q22.1	4	4
				shared	7q22.1-7q36.3	5	5
				shared	8p23.3-8q24.3	4	4
				shared	9p24.3-9q22.32	3	3
				shared	9q22.32-9q34.3	3	3
				shared	10q11.22	3	3
				shared	11p15.5-11p12	3	3
				shared	11p12-11p11.12	2 LOH	2 LOH
				shared	11p11.12-11q25	3	3
				shared	12p13.31-12p13.2	1	1
				shared	15	3	3
				shared	16q12.1-16q12.2	0	0
				shared	16q12.2	1	1
				shared	17p13.1	1	1
				shared	18	3	3
				shared	19p13.3-19p13.42	3	3
				shared	19q13.42-19q13.43	2 LOH	2 LOH
				shared	19q13.43	3	3
				shared	20	3	3
				shared	21	3	3
				unshared	12p13.33-12p13.31	1	2
				unshared	12p13.2-12p11.21	1	2
**46**	**Treated**	**Iliac crest left**	**L4**	shared	1p36.33-1q12	3	3
				shared	1q12-1q44	5	5
				shared	2	4	4
				shared	3	4	4
				shared	4	4	4
				shared	5	4	4
				shared	6	4	4
				shared	7	4	4
				shared	8	4	4
				shared	9	4	4
				shared	10p15.3-10q24.32	4	4
				shared	10q24.32	3	3
				shared	10q24.32-10q26.3	4	4
				shared	11	4	4
				shared	12	4	4
				shared	13	4	4
				shared	14	4	4
				shared	15	4	4
				shared	16p13.3	4	4
				shared	16p13.3	3	3

PID	Time point	RNAS	FNAS	Type	Region	Copy RNAS	Copy FNAS
				shared	16p13.3-16q22.1	4	4
				shared	16q22.1-16q22.2	3	3
				shared	16q22.2-16q24.3	4	4
				shared	17	4	4
				shared	18	4	4
				shared	19p13.3	6	6
				shared	19p13.3-19p13.2	5	5
				shared	19p13.2-19q13.33	4	4
				shared	19q13.33-19q13.43	3	3
				shared	20p13-20p11.1	4	4
				shared	20p11.1-20q11.21	6	6
				shared	20q11.21-20q13.33	4	4
				shared	21	4	4
				shared	22	4	4
				unshared	-	-	-
**47**	**Treated**	**Iliac crest left**	**Sacrum right**	shared (minor sub-clone in RNAS)	1p33-1p32.3	1	1
				shared	1p31.1-1p13.1	1	1
				shared	1p11.2-1q44	3	3
				shared	2q23.3-2q32.1	1	1
				shared	3q13.11-3q21.3	1	1
				shared	3q21.3-3q26.31	2 LOH	2 LOH
				shared	3q26.31-3q26.32	1	1
				shared	3q26.32	0	0
				shared	3q26.32-3q29	1	1
				shared	5q14.3-5q31.1	1	1
				shared	5q31.2-5q35.3	1	1
				shared	7p21.2-7p21.1	1	1
				shared	8p23.3-8p11.21	1	1
				shared	12p13.33-12p11.22	1	1
				shared	13	1	1
				shared	15	3	3
				shared	20p11.1-20q13.33	3	3
				shared	22	1	1
				unshared	19q11-19q13.2	1	2
**48**	**Treated**	**Iliac crest left**	**Ischium left**	shared	1q21.1-1q44	3	3
				shared	3q11.1-3q29	3	3
				shared	4q11-4q35.2	3	3
				shared	5	3	3
				shared	6p25.3-6q13	3	3
				shared	6q13-6q27	2 LOH	2 LOH
				shared	9q12-9q34.3	3	3
				shared	10q11.21-10q26.3	3	3

PID	Time point	RNAS	FNAS	Type	Region	Copy RNAS	Copy FNAS
				shared	11p15.5-11q22.3	3	3
				shared	11q23.1-11q23.3	3	3
				shared	11q23.3-11q24.2	4	4
				shared	11q24.2-11q24.3	3	3
				shared	11q24.3-11q25	2 LOH	2 LOH
				shared	11q25	3	3
				shared	13q11-13q13.3	1	1
				shared	13q14.11-13q34	1	1
				shared	14q32.33-14q32.33	1	1
				shared	15	3	3
				shared	19	4	4
				unshared	-	-	-
		**Iliac crest left**	**Ilium left**	shared	**Like ischium left**	-	-
				unshared	-	-	-
		**Iliac crest left**	**T8**	shared	**Like ischium left except chr 1, 6 and 19**		
				shared (minor sub-clone at T8)	6p25.3-6q13	3	3
				additional shared	19p13.3-19q13.43	4	4
				unshared	1q21.1-1q44	3	4
				unshared	17q12-17q25.3	2	3
				unshared	19p13.3	4	3
**49**	**Treated**	**Iliac crest left**	**Right chest wall mass**	shared	1p33-1p21.3	1	1
				shared	1q21.1	3 LOH	3 LOH
				shared	1q21.1-1q44	3	3
				shared	2	2 LOH	2 LOH
				shared	4q28.1-4q35.2	3	3
				shared	5p15.33-5p14.1	4	4
				shared	5p14.1-5p12	3	3
				shared	5p12-5q11.1	4	4
				shared	5q11.1-5q11.2	3	3
				shared	5q34-5q35.3	3	3
				shared	7p22.3	4	4
				shared	7q22.3-7q31.1	3	3
				shared	7q31.1-7q36.3	4	4
				shared	8q11.21-8q23.3	3	3
				shared	8q23.3-8q24.12	2 LOH	2 LOH
				shared	8q24.13-8q24.22	3	3
				shared	8q24.22	2 LOH	2 LOH
				shared	8q24.22-8q24.3	3	3
				shared	9p13.3-9p11.2	3	3
				shared	9q12-9q34.3	2 LOH	2 LOH

PID	Time point	RNAS	FNAS	Type	Region	Copy RNAS	Copy FNAS
				shared	12p13.33-12p11.1	4	4
				shared	12q11-12q24.33	3	3
				shared	13	1	1
				shared	14q32.33	1	1
				shared	15q11.1-15q14	3	3
				shared	19p13.3-19q12	3	3
				unshared	11q12.1-q12.3	2	3
**50**	**Treated**	**Iliac crest right**	**L2**	shared	1q21.3-1q22	3	3
				shared (minor sub-clone at L2)	2p25.3-2p23.3	1	1
				shared	4q31.3-4q35.2	3	3
				shared	5	3	3
				shared (minor sub-clone at L2)	6p24.3-6p21.1	3	3
				shared	7p22.3-7q21.12	3	3
				shared	7q21.12-7q21.13	4	4
				shared	7q21.13-7q36.3	3	3
				shared	8p23.3-8p21.1	1	1
				shared	9p24.3-9p11.2	4	4
				shared	11p11.12-11q25	3	3
				shared	15	4	4
				shared	18	1	1
				shared	19	3	3
				shared	21	3	3
				unshared	9q	4	3
				unshared	11p15.5-11p11.12	3	2
		**Iliac crest right**	**Ilium left**	shared	**Like L2 except chr9 and chr11**		
				additional shared	9	4	4
				additional shared	11	3	3
				unshared	-	-	-
**51**	**Treated**	**Iliac crest right**	**T12**	shared	1p32.3	1	1
				shared	2p16.2-2p16.1	1	1
				shared	2p12-2p11.2	1	1
				shared	2p11.2-2p11.1	2 LOH	2 LOH
				shared	3	3	3
				shared	4p16.3-4p15.2	1	1
				shared	5p15.33-5p15.31	3	3
				shared	6q14.1	1	1
				shared	6q15-6q16.1	1	1

PID	Time point	RNAS	FNAS	Type	Region	Copy RNAS	Copy FNAS
				shared	6q16.2-6q27	1	1
				shared	9p24.3-9p24.1	1	1
				shared	9p24.1	3	3
				shared	9p23	1	1
				shared	9p22.3-9p22.2	1	1
				shared	9p22.1-9p21.1	1	1
				shared	9p13.3-9p13.2	3	3
				shared	10q24.1-10q25.1	1	1
				shared	11p15.5-11q14.3	4	4
				shared	11q14.3-11q21	3	3
				shared	11q21-11q25	4	4
				shared	12p13.33-12p11.21	1	1
				shared	12q13.11-12q13.2	1	1
				shared	12q21.31-12q21.32	1	1
				shared	12q24.21-12q24.23	1	1
				shared	12q24.31-12q24.33	1	1
				shared	13q13.1-13q13.3	1	1
				shared	13q13.3-13q14.11	1	1
				shared	13q14.11	2 LOH	2 LOH
				shared	13q14.11-13q21.2	1	1
				shared	13q21.2	2 LOH	2 LOH
				shared	13q21.2-13q34	3	3
				shared	14q22.1-14q22.3	1	1
				shared	14q32.2	1	1
				shared	15q14-15q26.3	3	3
				shared	16p13.3-16p13.12	1	1
				shared	16p12.3-16p12.1	3	3
				shared	16p12.1	1	1
				shared	16q11.2-16q24.3	1	1
				shared	17p13.3-17q11.2	1	1
				shared	19p13.3-19p13.11	3	3
				shared	19p13.11-19q13.2	2 LOH	2 LOH
				shared	19q13.2-19q13.43	3	3
				shared	22q11.1-22q11.21	1	1
				shared	22q11.21-22q12.3	1	1
				shared	22q13.1-22q13.33	1	1
				unshared	-	-	-

Abbreviations: RNAS: random aspirate, FNAS: fine needle aspirate, Copy: copy number at indicated region; LOH: loss of heterozygosity
