# Supplementary material for: Spatial genomic heterogeneity in multiple myeloma revealed by multi-region sequencing
Source: Nat Commun. 2017 Aug 16;8:268. doi: 10.1038/s41467-017-00296-y (PMC5559527; doi:10.1038/s41467-017-00296-y)
Supplement: Supplementary file 4 — Supplementary Data 3 [file 41467_2017_296_MOESM4_ESM.pdf]

Supplementary Data 3: Number of non-silent mutations.

PID	Time	Total mutations [≥5% VAF]	Sub-clonal unshared mutations [0.20≤CCF<0.80]	Clonal unshared mutations [CCF≥0.80]	Shared-diff mutations [CCF≥0.20]
1	Baseline	56	9	20	15
2	Baseline	50	18	4	14
3	Baseline	45	18	5	6
4	Baseline	113	35	31	5
5	Baseline	57	6	4	15
6	Baseline	46	11	4	5
7	Baseline	49	10	11	0
8	Baseline	59	4	20	1
9	Baseline	46	5	1	13
10	Baseline	56	7	0	14
11	Baseline	46	14	0	3
12	Baseline	55	4	14	2
13	Baseline	59	5	0	15
14	Baseline	65	7	6	9
15	Baseline	62	16	3	0
16	Baseline	46	6	2	3
17	Baseline	58	8	0	5
18	Baseline	50	5	4	1
19	Baseline	120	11	11	2
20	Baseline	55	6	4	0
21	Baseline	20	1	0	2
22	Baseline	435	59	0	5
23	Baseline	41	5	1	0
24	Baseline	50	2	0	5
25	Baseline	40	0	0	5
26	Baseline	19	1	0	1
27	Baseline	51	2	0	2
28	Baseline	72	2	2	1
29	Baseline	87	4	0	2
30	Baseline	34	2	0	0
31	Baseline	94	1	0	4
32	Baseline	40	0	0	2
33	Baseline	41	1	0	1
34	Baseline	97	3	0	0
35	Baseline	37	0	0	1
36	Baseline	63	0	0	1
37	Baseline	30	0	0	0
38	Baseline	21	0	0	0
39	Baseline	84	0	0	0
40	Baseline	34	0	0	0
41	Baseline	46	0	0	0
42	Baseline	47	0	0	0

PID	Time	Total mutations [≥5% VAF]	Sub-clonal unshared mutations [0.20≤CCF<0.80]	Clonal unshared mutations [CCF≥0.80]	Shared-diff mutations [CCF≥0.20]
28	Treated	82	6	0	1
32	Treated	33	3	0	0
43	Treated	97	26	15	25
44	Treated	79	7	35	3
45	Treated	58	9	0	0
46	Treated	186	5	0	15
47	Treated	52	4	0	0
48	Treated	400	11	12	1
49	Treated	78	2	0	1
50	Treated	40	0	0	1
51	Treated	104	0	0	0

Abbreviations: VAF: variant allele frequency; CCF: cancer clonal fraction
