# Supplementary material for: Spatial genomic heterogeneity in multiple myeloma revealed by multi-region sequencing
Source: Nat Commun. 2017 Aug 16;8:268. doi: 10.1038/s41467-017-00296-y (PMC5559527; doi:10.1038/s41467-017-00296-y)
Supplement: Supplementary file 5 — Supplementary Data 4 [file 41467_2017_296_MOESM5_ESM.pdf]

Supplementary Data 4: Previous therapies for “Treated patients”

PID	Diagnosis (year)	Previous Therapies
28	2014	up-front metronomic therapy
32	2014	2 x MVDT PACE
43	2003	DT-PACE<br>Melphalan with ASCT<br>BEAM with ASCT<br>DT-PACE consolidation<br>Thal/Dex<br>*1. Relapse*<br>VTD<br>*2. Relapse*<br>Pom<br>*3. Relapse*<br>CFZ/Dex<br>*4. Relapse*<br>metronomic, Arsenic trioxide
44	2012	Radiation therapy<br>MVTD PACE<br>Melphalan with ASCT<br>VRD
45	2009	VTD<br>VTDPACE<br>1. Relapse<br>CFZ/Dex<br>CRD<br>Progression<br>CFZ/Arsenic/Pom/Atra/Dex
46	2003	DT-PACE<br>2 x Melphalan with ASCT<br>VTD consolidation
47	2011	MVTD PACE<br>2 x Melphalan with ASCT<br>VRD consolidation
48	2012	VRD<br>Radiation therapy<br>CFZ/Dex
49	2009	MVTD PACE<br>Melphalan with ASCT<br>VRD consolidation<br>1.Relapse<br>Metronomic therapy<br>2. Relapse<br>Trametinib<br>3. Relapse:<br>Trametinib/Pom/Dex
50	2006	DT-PACE<br>Melphalan/VTD + ASCT<br>Thal<br>VDTPACE consolidation

PID	Diagnosis (year)	Previous Therapies
		Thal<br>1. Relapse<br>VDT<br>Len/Dex
51	2004	VDT-PACE<br>2 x Melphalan with ASCT<br>VTD<br>Thal 50mg + Dex 20mg<br>Len

Abbreviations: Dex: Dexamethasone; (V)DTPAC(E): (Bortezomib) Dexamethasone, Thalidomide, Cisplatin, Doxorubicin, Cyclophasphamide, (Etoposide); ASCT: autologous stem cell transplantation; Pom: Pomalidomide; CFZ: Carfilzomib; Metronomic therapy:, Doxorubicin&Cisplatin as continuous 24-h intravenous infusion for 16 days plus Bortezomib, Thalidomide: Dexamethasone; Len: Lenalidomide; MVDT: Melphalan/VTD; CRD: Carfilzomib, Lenalidomide, Dexamethasone
