# Supplementary material for: Spatial genomic heterogeneity in multiple myeloma revealed by multi-region sequencing
Source: Nat Commun. 2017 Aug 16;8:268. doi: 10.1038/s41467-017-00296-y (PMC5559527; doi:10.1038/s41467-017-00296-y)
Supplement: Supplementary file 6 — Supplementary Data 5 [file 41467_2017_296_MOESM6_ESM.pdf]

Supplementary Data 5: Identification of evolutionary dynamics in multiple myeloma.

| PID | Type | Mutations [≥5% VAF] | Mutations [VAF 0.12-0.24] | $R^2$ | Unshared mutations [CCF≥0.8] | Shared-diff mutations [CCF≥0.8] |
|---|---|---|---|---|---|---|
| 1 | FNAS | 90 | 5 | - | 25 | 27 |
| 1 | RNAS | 48 | 5 | - | 7 | 0 |
| 2 | FNAS | 71 | 7 | - | 2 | 33 |
| 2 | RNAS | 89 | 15 | 0.92 | 8 | 0 |
| 3 | FNAS | 49 | 2 | - | 12 | 10 |
| 3 | RNAS | 57 | 29 | 0.94 | 0 | 0 |
| 4 | FNAS | 143 | 25 | 0.57 | 54 | 11 |
| 4 | RNAS | 128 | 36 | 0.87 | 7 | 0 |
| 5 | FNAS | 128 | 15 | 0.92 | 9 | 26 |
| 5 | RNAS | 54 | 10 | - | 0 | 0 |
| 6 | FNAS | 53 | 7 | - | 6 | 8 |
| 6 | RNAS | 63 | 13 | 0.92 | 0 | 0 |
| 7 | FNAS 1 | 44 | 3 | - | 8 | 0 |
| 7 | FNAS 2 | 44 | 9 | - | 9 | 0 |
| 7 | RNAS | 45 | 5 | - | 1 | 0 |
| 8 | FNAS 1 | 59 | 0 | - | 3 | 0 |
| 8 | FNAS 2 | 63 | 1 | - | 11 | 1 |
| 8 | FNAS 3 | 75 | 1 | - | 11 | 1 |
| 8 | RNAS | 69 | 5 | - | 6 | 0 |
| 9 | FNAS | 64 | 14 | 0.88 | 1 | 1 |
| 9 | RNAS | 61 | 7 | - | 0 | 0 |
| 10 | FNAS | 85 | 20 | 0.97 | 0 | 4 |
| 10 | RNAS | 63 | 5 | - | 1 | 3 |
| 11 | FNAS | 82 | 19 | 0.96 | 0 | 0 |
| 11 | RNAS | 60 | 13 | 0.68 | 0 | 0 |
| 12 | FNAS 1 | 64 | 4 | - | 14 | 3 |
| 12 | FNAS 2 | 62 | 4 | - | 9 | 1 |
| 12 | FNAS 3 | 62 | 5 | - | 9 | 2 |
| 12 | FNAS 4 | 64 | 5 | - | 10 | 3 |
| 12 | RNAS | 63 | 25 | 0.64 | 5 | 0 |
| 13 | FNAS | 68 | 2 | - | 0 | 5 |
| 13 | RNAS | 85 | 15 | 0.8 | 0 | 5 |
| 14 | FNAS | 78 | 0 | - | 12 | 10 |
| 14 | RNAS | 88 | 28 | 0.9 | 0 | 0 |
| 15 | FNAS 1 | 68 | 6 | - | 4 | 3 |
| 15 | FNAS 2 | 69 | 9 | - | 2 | 2 |
| 15 | RNAS | 95 | 27 | 0.97 | 0 | 0 |
| 16 | FNAS | 62 | 3 | - | 3 | 3 |
| 16 | RNAS | 77 | 6 | - | 0 | 0 |
| 17 | FNAS | 99 | 13 | 0.95 | 0 | 0 |
| 17 | RNAS | 56 | 28 | 0.89 | 0 | 0 |
| 18 | FNAS | 68 | 7 | - | 9 | 0 |

| PID | Type | Mutations [≥5% VAF] | Mutations [VAF 0.12-0.24] | $R^2$ | Unshared mutations [CCF≥0.8] | Shared-diff mutations [CCF≥0.8] |
|---|---|---|---|---|---|---|
| 18 | RNAS | 62 | 12 | 0.73 | 0 | 0 |
| 19 | FNAS | 205 | 8 | - | 22 | 7 |
| 19 | RNAS | 191 | 22 | 0.75 | 5 | 0 |
| 20 | FNAS 1 | 88 | 15 | 0.64 | 6 | 2 |
| 20 | FNAS 2 | 93 | 13 | 0.87 | 0 | 0 |
| 20 | FNAS 3 | 86 | 8 | - | 0 | 0 |
| 20 | RNAS | 90 | 8 | - | 0 | 0 |
| 21 | FNAS | 29 | 2 | - | 0 | 0 |
| 21 | RNAS | 31 | 1 | - | 0 | 0 |
| 22 | FNAS | 773 | 463 | 0.87 | 0 | 0 |
| 22 | RNAS | 716 | 65 | 0.9 | 0 | 0 |
| 23 | FNAS | 71 | 3 | - | 2 | 0 |
| 23 | RNAS | 77 | 13 | 0.72 | 0 | 0 |
| 24 | FNAS | 80 | 13 | 0.8 | 0 | 0 |
| 24 | RNAS | 80 | 9 | - | 0 | 2 |
| 25 | FNAS 1 | 72 | 8 | - | 0 | 0 |
| 25 | FNAS 2 | 70 | 16 | 0.93 | 0 | 0 |
| 25 | FNAS 3 | 71 | 17 | 0.92 | 0 | 0 |
| 25 | RNAS | 66 | 12 | 0.9 | 0 | 0 |
| 26 | FNAS | 33 | 2 | - | 0 | 4 |
| 26 | RNAS | 35 | 1 | - | 0 | 0 |
| 27 | FNAS | 77 | 18 | 0.91 | 0 | 0 |
| 27 | RNAS | 74 | 13 | 0.97 | 0 | 0 |
| 28 | FNAS 1 | 50 | 2 | - | 1 | 1 |
| 28 | FNAS 2 | 50 | 0 | - | 1 | 0 |
| 28 | RNAS | 46 | 3 | - | 0 | 0 |
| 29 | FNAS | 166 | 27 | 0.76 | 0 | 0 |
| 29 | RNAS | 141 | 36 | 0.78 | 0 | 0 |
| 30 | FNAS | 63 | 2 | - | 0 | 1 |
| 30 | RNAS | 73 | 5 | - | 1 | 1 |
| 31 | FNAS 1 | 87 | 3 | - | 0 | 0 |
| 31 | FNAS 2 | 165 | 6 | - | 0 | 0 |
| 31 | FNAS 3 | 144 | 6 | - | 0 | 0 |
| 31 | RNAS | 99 | 6 | - | 0 | 0 |
| 32 | FNAS | 56 | 3 | - | 0 | 0 |
| 32 | RNAS | 71 | 14 | 0.73 | 0 | 0 |
| 33 | FNAS | 66 | 4 | - | 0 | 0 |
| 33 | RNAS | 62 | 28 | 0.85 | 0 | 0 |
| 34 | FNAS | 124 | 4 | - | 0 | 0 |
| 34 | RNAS | 146 | 11 | - | 0 | 0 |
| 35 | FNAS 1 | 57 | 7 | - | 0 | 0 |
| 35 | FNAS 2 | 55 | 8 | - | 0 | 0 |

| PID | Type | Mutations [≥5% VAF] | Mutations [VAF 0.12-0.24] | $R^2$ | Unshared mutations [CCF≥0.8] | Shared-diff mutations [CCF≥0.8] |
|---|---|---|---|---|---|---|
| 35 | RNAS | 55 | 7 | - | 0 | 0 |
| 36 | FNAS | 131 | 0 | - | 0 | 4 |
| 36 | RNAS | 134 | 7 | - | 0 | 0 |
| 37 | FNAS | 43 | 2 | - | 0 | 0 |
| 37 | RNAS | 45 | 2 | - | 0 | 0 |
| 38 | FNAS | 37 | 3 | - | 0 | 0 |
| 38 | RNAS | 38 | 6 | - | 0 | 0 |
| 39 | FNAS | 160 | 2 | - | 0 | 0 |
| 39 | RNAS | 149 | 1 | - | 0 | 0 |
| 40 | FNAS | 61 | 1 | - | 0 | 0 |
| 40 | RNAS | 66 | 8 | - | 0 | 0 |
| 41 | FNAS | 70 | 15 | 0.69 | 1 | 0 |
| 41 | RNAS | 64 | 13 | 0.69 | 0 | 0 |
| 42 | FNAS | 81 | 16 | 0.78 | 1 | 0 |
| 42 | RNAS | 86 | 19 | 0.88 | 0 | 0 |

Abbreviations: RNAS: random aspirate, FNAS: fine needle aspirate, VAF: variant allele frequency,
CCF: cancer clonal fraction, $R^2$: goodness-of-fit measure for cumulative distribution of subclonal mutations
