# Supplementary material for: Spatial genomic heterogeneity in multiple myeloma revealed by multi-region sequencing
Source: Nat Commun. 2017 Aug 16;8:268. doi: 10.1038/s41467-017-00296-y (PMC5559527; doi:10.1038/s41467-017-00296-y)
Supplement: Supplementary file 7 — Supplementary Data 6 [file 41467_2017_296_MOESM7_ESM.pdf]

**Supplementary Data 6: Confirmation of unshared mutations by deep whole exome sequencing.**

| PID | Mutation | Symbol | Effect | Change | CCF | Reads | Alternative reads |
|---|---|---|---|---|---|---|---|
| 3 | 1:11169374T>A | MTOR | missense | Ile2501Phe | 0.58 | 316 | not detectable |
| 3 | 1:38484759A>G | UTP11L | missense | His124Arg | 0.55 | 328 | not detectable |
| 3 | 1:112525054G>A | KCND3 | missense | Arg99Cys | 1.00 | 896 | not detectable |
| 3 | 2:179401215C>T | TTN | missense | Gly33420Glu | 0.80 | 629 | 4 |
| 3 | 3:78708951C>T | ROBO1 | missense | Ser776Asn | 0.70 | 304 | not detectable |
| 3 | 4:96046159C>T | BMPR1B | stop_gained | Arg188* | 0.48 | 329 | not detectable |
| 3 | 7:156762366G>A | NOM1 | missense | Cys851Tyr | 0.94 | 193 | not detectable |
| 3 | 8:86048171G>A | LRRCC1 | missense | Val768Ile | 0.58 | 221 | not detectable |
| 3 | 8:145011190T>C | PLEC | missense | Lys268Arg | 0.30 | 1068 | not detectable |
| 3 | 9:116811731C>T | ZNF618 | missense | Arg717Trp | 0.58 | 833 | not detectable |
| 3 | 10:89621877C>A | KLLN | missense | Arg123Leu | 1.02 | 665 | not detectable |
| 3 | 11:1756516G>T | IFITM10 | missense | Asp227Glu | 0.50 | 597 | not detectable |
| 3 | 11:106888683C>G | GUCY1A2 | missense | Arg33Ser | 0.39 | 510 | not detectable |
| 3 | 12:52284640G>A | ANKRD33 | missense | Val304Ile | 0.60 | 795 | not detectable |
| 3 | 12:53493080C>A | IGFBP6 | missense | Leu122Ile | 1.16 | 394 | not detectable |
| 3 | 14:47426708C>T | MDGA2 | missense | Arg584Gln | 0.52 | 305 | not detectable |
| 3 | 17:42882299G>T | GJC1 | missense | Pro296Gln | 0.32 | 546 | not detectable |
| 3 | 17:74076670C>T | ZACN | missense | Leu237Phe | 0.40 | 969 | not detectable |
| 3 | 17:76127689T>A | TMC8 | missense | Val7Glu | 0.32 | 240 | not detectable |
| 3 | 18:12344148C>T | AFG3L2 | missense | Ala588Thr | 0.68 | 544 | not detectable |
| 3 | 19:9074202G>C | MUC16 | missense | Thr4415Ser | 0.38 | 489 | not detectable |
| 3 | 21:46875781G>A | COL18A1 | missense | Glu113Lys | 0.66 | 1039 | 2 |
| 3 | 22:50312874G>T | CRELD2 | splice_region | | 0.32 | 473 | not detectable |
| 7 | 1:44878074C>A | RNF220 | missense | Pro102Gln | 0.79 | 554 | not detectable |
| 7 | 1:115252204C>T | NRAS | missense | Ala146Thr | 0.88 | 263 | not detectable |
| 7 | 1:117208939A>T | IGSF3 | missense | Phe4Ile | 0.83 | 248 | not detectable |
| 7 | 1:163138061C>T | RGS5 | missense | Ala68Thr | 0.81 | 417 | not detectable |
| 7 | 1:222827749G>A | MIA3 | missense | Val1466Ile | 0.79 | 297 | not detectable |
| 7 | 2:42936062C>T | MTA3 | missense | Pro451Ser | 0.20 | 308 | not detectable |
| 7 | 2:179605986C>T | TTN | missense | Gly3992Ser | 0.96 | 558 | not detectable |
| 7 | 5:7626355C>T | ADCY2 | missense | Leu216Phe | 0.76 | 512 | not detectable |
| 7 | 5:176562325G>C | NSD1 | missense | Arg74Thr | 0.62 | 909 | not detectable |
| 7 | 9:125622407T>A | RC3H2 | missense | Lys546Asn | 1.17 | 417 | not detectable |
| 7 | 10:88486007G>A | LDB3 | missense | Ala703Thr | 0.63 | 529 | not detectable |
| 7 | 12:57974872G>A | KIF5A | missense | Arg891His | 0.95 | 403 | not detectable |
| 7 | 12:106715333G>T | TCP11L2 | missense | Asp162Tyr | 0.89 | 320 | 2 |
| 7 | 14:20843997T>G | TEP1 | missense | Lys2094Gln | 0.85 | 263 | not detectable |
| 7 | 14:93760431C>A | BTBD7 | missense | Arg312Ile | 1.00 | 250 | not detectable |
| 7 | 15:38235541G>A | TMCO5A | splice_acceptor | | 0.71 | 409 | not detectable |
| 7 | 15:42704306G>A | CAPN3 | missense | Arg156Gln | 0.68 | 316 | 2 |
| 7 | 17:7345197C>A | FGF11 | stop_gained | Ser136* | 0.76 | 394 | not detectable |
| 7 | 17:48650034G>A | CACNA1G | missense | Arg289His | 0.92 | 314 | not detectable |
| 7 | 19:11030322A>C | CARM1 | missense | Asn358His | 0.30 | 488 | not detectable |
| 7 | 19:40419732C>T | FCGBP | missense | Val1088Met | 1.09 | 305 | not detectable |
| 8 | 1:182026286GCCTTC>G | ZNF648 | frameshift | Lys286fs | 0.91 | 303 | 3 |

| PID | Mutation | Symbol | Effect | Change | CCF | Reads | Alternative reads |
|---|---|---|---|---|---|---|---|
| 8 | 1:247902614C>T | OR14K1 | missense | Ser233Phe | 1.03 | 1180 | not detectable |
| 8 | 2:3196197C>T | TSSC1 | missense | Arg353His | 0.85 | 1064 | not detectable |
| 8 | 2:141116507G>A | LRP1B | missense | Leu3714Phe | 0.96 | 510 | not detectable |
| 8 | 2:208632713A>T | FZD5 | missense | Ser251Thr | 0.89 | 1311 | not detectable |
| 8 | 3:17255764CT>C | TBC1D5 | frameshift | Asp585fs | 0.88 | 690 | not detectable |
| 8 | 3:191888370A>G | FGF12 | missense | Tyr164His | 1.11 | 662 | not detectable |
| 8 | 6:166307823C>CA | SDIM1 | frameshift | Ala111fs | 0.49 | 737 | not detectable |
| 8 | 7:129098087T>G | STRIP2 | missense | Ile357Ser | 1.00 | 897 | not detectable |
| 8 | 7:140453145A>C | BRAF | missense | Leu597Arg | 0.82 | 516 | not detectable |
| 8 | 10:98144406C>T | TLL2 | missense | Arg711His | 0.36 | 645 | not detectable |
| 8 | 10:115970624C>T | TDRD1 | missense | Leu520Phe | 0.83 | 701 | not detectable |
| 8 | 11:3125479C>T | OSBPL5 | missense | Arg112His | 1.01 | 1125 | not detectable |
| 8 | 11:70331819C>T | SHANK2 | missense | Val1528Met | 0.87 | 1107 | not detectable |
| 8 | 12:25398284C>A | KRAS | missense | Gly12Val | 0.91 | 484 | not detectable |
| 8 | 12:58157886T>G | CYP27B1 | missense | Lys404Gln | 1.09 | 1091 | not detectable |
| 8 | 14:45414708G>A | KLHL28 | missense | Arg156Cys | 0.73 | 504 | not detectable |
| 8 | 14:103173833A>T | RCOR1 | missense | His215Leu | 0.95 | 352 | not detectable |
| 8 | 17:3030793G>A | OR1G1 | missense | Ser18Phe | 0.99 | 585 | not detectable |
| 8 | 17:40474420C>A | STAT3 | missense | Asp661Tyr | 0.91 | 938 | not detectable |
| 8 | 17:40475367G>T | STAT3 | missense | Asn553Lys | 0.93 | 634 | not detectable |
| 8 | 19:43708102T>A | PSG4 | missense | Leu122Phe | 0.94 | 1398 | not detectable |
| 8 | 19:56545076G>A | NLRP5 | splice_donor | | 1.00 | 702 | not detectable |
| 8 | 20:37662863A>T | DHX35 | missense | Glu657Val | 0.73 | 509 | not detectable |
| 19 | 1:64120033C>T | PGM1 | stop_gained | Arg517* | 0.70 | 425 | not detectable |
| 19 | 2:86302386G>T | POLR1A | splice_region | | 0.70 | 248 | not detectable |
| 19 | 2:98851110G>C | VWA3B | splice_acceptor | | 0.34 | 193 | not detectable |
| 19 | 2:242438827C>T | STK25 | missense | Ala144Thr | 0.82 | 345 | not detectable |
| 19 | 3:38892034T>C | SCN11A | missense | Gln1422Arg | 0.67 | 521 | not detectable |
| 19 | 3:130311929G>C | COL6A6 | missense | Asp1466His | 0.29 | 463 | not detectable |
| 19 | 4:141560502A>C | TBC1D9 | missense | Asp806Glu | 0.96 | 229 | not detectable |
| 19 | 5:131607495G>T | PDLIM4 | missense | Gly228Trp | 0.97 | 547 | not detectable |
| 19 | 5:140516485T>A | PCDHB5 | missense | Leu490Gln | 1.07 | 346 | not detectable |
| 19 | 5:149214922A>G | PPARGC1B | missense | Thr601Ala | 0.26 | 418 | not detectable |
| 19 | 5:179306693C>T | TBC1D9B | missense | Ala451Thr | 0.82 | 273 | not detectable |
| 19 | 7:87051441A>C | ABCB4 | missense | Leu771Arg | 0.85 | 166 | not detectable |
| 19 | 7:107872789G>T | NRCAM | missense | Asn136Lys | 0.91 | 388 | not detectable |
| 19 | 10:104158183G>C | NFKB2 | missense | Lys298Asn | 0.24 | 604 | not detectable |
| 19 | 11:67378589T>G | NDUFV1 | missense | Leu275Arg | 0.91 | 919 | not detectable |
| 19 | 16:1245505G>C | CACNA1H | missense | Gly162Ala | 0.92 | 402 | not detectable |
| 19 | 17:38285680C>T | MSL1 | missense | Ser392Phe | 0.47 | 193 | not detectable |
| 19 | 18:8796244C>T | SOGA2 | missense | Pro489Ser | 0.91 | 253 | not detectable |
| 19 | 19:1627421C>T | TCF3 | splice_acceptor | Lys50Lys | 1.03 | 378 | not detectable |
| 19 | 19:48821678T>A | CCDC114 | missense | Glu72Val | 0.67 | 489 | not detectable |
| 19 | 19:52394982C>T | ZNF649 | missense | Gly136Glu | 0.23 | 599 | not detectable |
| 19 | 19:54598512G>A | OSCAR | missense | Arg260Cys | 0.23 | 326 | not detectable |

Abbreviations: PID: patient identification number, CCF: cancer clonal fraction in positive sample,

reads: total number of reads at respective site in negative sample,
alternative reads: number of reads indicating mutation at negative site (detection threshold of 2 reads).
